# Preclinical Evaluation of a Lentiviral Vector for Huntingtin Silencing

**DOI:** 10.1016/j.omtm.2017.05.001

**Published:** 2017-05-11

**Authors:** Karine Cambon, Virginie Zimmer, Sylvain Martineau, Marie-Claude Gaillard, Margot Jarrige, Aurore Bugi, Jana Miniarikova, Maria Rey, Raymonde Hassig, Noelle Dufour, Gwenaelle Auregan, Philippe Hantraye, Anselme L. Perrier, Nicole Déglon

**Affiliations:** 1 CEA, DRF, Institute of Biology Francois Jacob, Molecular Imaging Research Center, F-92265 Fontenay-aux-Roses, France; 2 CNRS, CEA, Paris-Sud University, University Paris-Saclay, Neurodegenerative Diseases Laboratory (UMR9199), F-92265 Fontenay-aux-Roses, France; 3 Department of Clinical Neurosciences, Laboratory of Cellular and Molecular Neurotherapies, Lausanne University Hospital (CHUV), 1011 Lausanne, Switzerland; 4 Neuroscience Research Center, Laboratory of Cellular and Molecular Neurotherapies, Lausanne University Hospital (CHUV), 1011 Lausanne, Switzerland; 5 Department of Research & Development, uniQure, 1105 Amsterdam, the Netherlands; 6 Institut National de la Santé et de la Recherche Médicale UMR861, I-Stem, AFM, 91100 Corbeil-Essonnes, France; 7 UEVE UMR861, I-STEM, AFM, 91100 Corbeil-Essonnes, France; 8 CECS, I-STEM, AFM, 91100 Corbeil-Essonnes, France

**Keywords:** Huntington, RNA interference, biosafety, off-targets, gene expression, iPSCs

## Abstract

Huntington’s disease (HD) is an autosomal dominant neurodegenerative disorder resulting from a polyglutamine expansion in the huntingtin (HTT) protein. There is currently no cure for this disease, but recent studies suggest that RNAi to downregulate the expression of both normal and mutant HTT is a promising therapeutic approach. We previously developed a small hairpin RNA (shRNA), vectorized in an HIV-1-derived lentiviral vector (LV), that reduced pathology in an HD rodent model. Here, we modified this vector for preclinical development by using a tat-independent third-generation LV (pCCL) backbone and removing the original reporter genes. We demonstrate that this novel vector efficiently downregulated *HTT* expression in vitro in striatal neurons derived from induced pluripotent stem cells (iPSCs) of HD patients. It reduced two major pathological HD hallmarks while triggering a minimal inflammatory response, up to 6 weeks after injection, when administered by stereotaxic surgery in the striatum of an in vivo rodent HD model. Further assessment of this shRNA vector in vitro showed proper processing by the endogenous silencing machinery, and we analyzed gene expression changes to identify potential off-targets. These preclinical data suggest that this new shRNA vector fulfills primary biosafety and efficiency requirements for further development in the clinic as a cure for HD.

## Introduction

Huntington’s disease (HD) (MIM 143100) is a neurodegenerative disorder inherited in an autosomal dominant manner marked by progressive loss of neurons, particularly in the striatum. The HD-causing mutation is a CAG repeat expansion located in exon 1 of the huntingtin (*HTT*) gene that is translated into an expanded polyglutamine stretch in the mutant-HTT isoform.^1^, ^2^ The HTT protein is involved in neurogenesis and the specification of neuronal and non-neuronal lineages,^3^, ^4^ vesicle transport,^5^, ^6^, ^7^ potassium-based cyclic AMP (cAMP) chemotaxis,^8^ and interactions with transcription factors.^9^ HD initially results in involuntary choreic movements that progressively evolve toward rigidity and dystonia, cognitive impairment with dementia, and neuropsychiatric deficits, leading to death after 15–20 years. There is currently no cure or way to stop the progression of HD. Thus, patient care focuses on the management of symptoms and improvement of the quality of life.^10^, ^11^, ^12^, ^13^ The HD mutation is an obvious target for therapeutic intervention based on gene silencing or genome editing, because it is at the top of all pathological cascades. The most pragmatic approach, supported by the dominant nature of HD inheritance, consists, however, of silencing both alleles of the *HTT* gene. *HTT* gene silencing in the brain reduces *HTT* transcript levels, decreases the formation of inclusions, and improves behavioral deficits in HD animal models.^14^, ^15^, ^16^, ^17^ Studies in *HTT* knockout (KO) mice clearly demonstrate that HTT is important for embryonic development.^18^, ^19^, ^20^ However, HTT depletion in the adult brain (after 4 months of age)^21^ and inhibition of wild-type (WT) HTT expression in rodents does not appear to cause detectable dysfunction, suggesting that the risk/benefit ratio of long-term HTT silencing in the adult human brain would favor the lowering of both WT and mutant HTT isoforms in HD patients.^17^, ^22^, ^23^, ^24^

RNAi is based on naturally occurring and conserved molecular machinery that induces gene silencing.^25^ Exogenous and artificial RNAi (small hairpin RNA [shRNA]) are vectorized to ensure continuous and long-term expression in the CNS.^26^ Potency and specificity of an shRNA depend on multiple parameters, including target mRNA abundance,^27^, ^28^, ^29^, ^30^, ^31^, ^32^ turnover or cellular localization,^33^ the presence of specific RNA binding proteins,^34^ and the structure, length, and internal stability of the shRNA itself.^35^, ^36^, ^37^, ^38^, ^39^

Mechanistic studies of vectorized shRNA have paved the way for powerful preclinical proof-of-concept studies that support the therapeutic relevance of HTT-lowering strategies.^14^, ^15^, ^16^, ^17^, ^22^, ^23^, ^24^, ^40^, ^41^, ^42^, ^43^, ^44^, ^45^, ^46^, ^47^, ^48^, ^49^ However, assessment of the biosafety of the gene therapy product is still a major challenge for clinical application in HD. Issues raised by shRNA include the potential lack of specificity (e.g., large number of off-target mRNA molecules can create a “dilution effect” that limits on-target shRNA-lowering activity^30^, ^32^), adverse effects caused by lowering the expression of off-target genes, cytotoxicity of the vehicle itself^50^ (e.g., through the interferon response),^51^, ^52^, ^53^ or overload of the cellular microRNA (miRNA) machinery, in particular of Exportin-5 and Argonaute-2 by excessive shRNA production (e.g., when the shRNA is driven by a strong polymerase II promoter).^54^

In a previous study in rodents, we developed a lentiviral vector expressing an shRNA targeting a sequence common to human and mouse in exons 3 and 4 of *HTT*, named shHTT6.^17^ The 19 nt passenger and guide strand are connected by a 9 nt loop and are expressed from the H1 polymerase III promoter.^17^, ^26^ This shRNA was designed to have the passenger strand at the 5′ end of the hairpin (right-hand loop, R-type shRNAs).^55^, ^56^ The H1 driving shHTT6 expression is active both in neurons and in astrocytes, two cell types affected in HD. H1-shHTT6 was originally cloned into the 3′ self-inactivating (SIN)-LTR (long terminal repeat) of an HIV-1-derived lentiviral vector (LV) containing a reporter gene (LacZ or GFP). This design was ideal for facilitating the identification of transduced cells for experimental purposes. Here, we selected a third-generation tat-independent LV (pCCL) backbone^57^ for preclinical development and further address the biosafety profile of this vector in neurons derived from HD-patient-specific induced pluripotent stem cells (iPSCs), following administration of LV-shHTT6 in the striatum of rodent.

## Results

### Development of a Third-Generation LV Encoding shRNA Targeting the Human WT and Mutant *HTT* and Evaluation of Silencing Efficiency In Vitro

**Figure 1:**
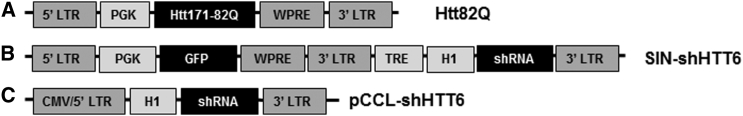
Schematic Representation of LVs Used in the Study (A) Lentiviral vector used to overexpress the first 171 (htt171-82Q) amino acids of the HTT protein with 82 CAG. (B) Second-generation lentiviral vector used to encode shHTT6. Here, the H1-shHTT6 was cloned into the 3′ SIN LTR of an HIV-1 LV (called SIN-shHTT6) including a GFP reporter gene to allow the tracking of infected cells (C). For clinical purposes, the third-generation LV (called pCCL-shHTT6), encoding the same shHTT6, is cloned in a tat-independent pCCL backbone.

**Figure 2:**
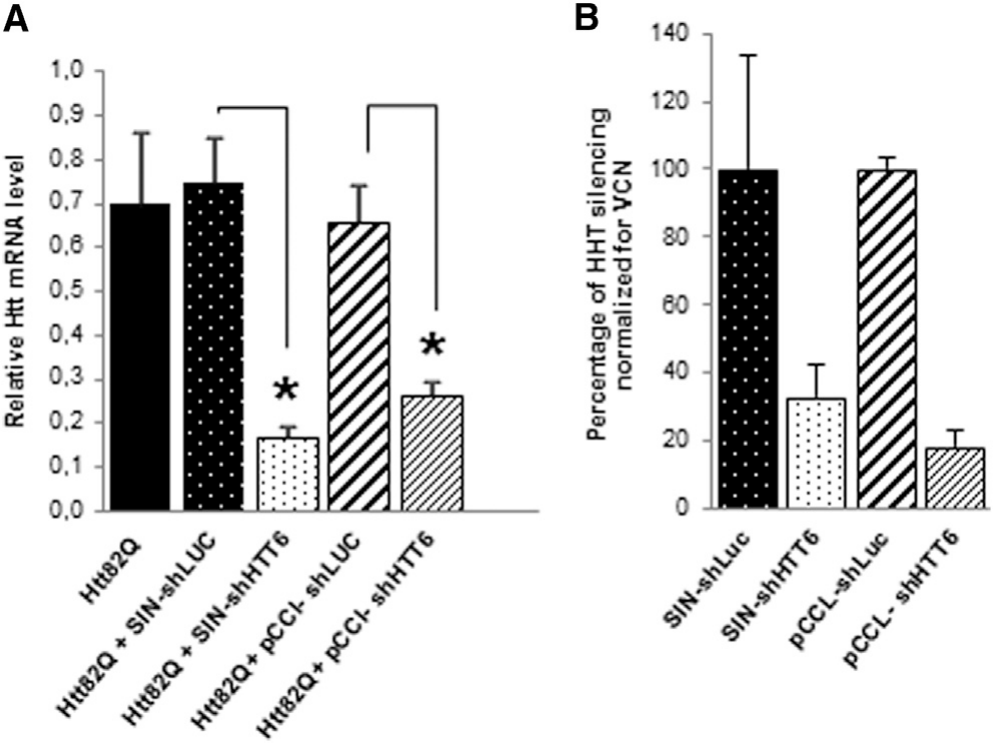
Evaluation of the Efficiency of HTT Silencing by SIN and pCCL-shHHT6 LVs In Vitro (A) 293T cells were co-infected with 150 ng of LVs encoding Htt171-82Q and shHTT6 or their respective negative control shRNA targeting luciferase, and cultured for 5 days. qPCR analyses show that all vectors efficiently silenced the expression of HTT. All values were normalized to β-actin and expressed as mean values ± SEM (n = 3–4 for each group) (Mann-Whitney *U* test, *p < 0.05). (B) Normalization of HTT expression by VCN shows similar levels of endogenous WT HTT silencing in 293T cells infected with either SIN-shHTT6 or pCCL-shHTT6 (chi-square test, df = 47.54, 6; p < 0.0001). A representative result of three independent experiments is shown.

The expression cassette containing the H1 polymerase III promoter and the shHTT6 target sequence was cloned into the lentiviral pCCL backbone^57^ (hereafter named pCCL-shHTT6) (Figure 1). We evaluated the ability of pCCL-shHTT6 to silence human *HTT* expression in vitro by qRT-PCR of infected HEK293T cells. The cells were co-infected with a vector encoding the first 171 amino acids of human HTT with 82 polyglutamines (Htt171-82Q) and the LV (SIN and pCCL backbones) directed against the luciferase reporter gene (shRNA targeting the luciferase mRNA [shLUC]) or shHTT6. The pCCL-shHTT6 reduced human mutant *HTT* expression by 62% relative to cells infected with Htt171-82Q combined with the corresponding pCCL-shLUC (Mann-Whitney *U* test, p < 0.05). The SIN-shHtt6 vector decreased *HTT* expression by 75.9% ± 14.0% (Mann-Whitney *U* test, p < 0.05), consistent with our previous studies^17^ (Figure 2A). We further compared the relative efficacy of these two constructs by performing additional experiments in HEK293T cells, which express WT human HTT. We quantified and used the number of integrated copies of LV for the normalization of *HTT* silencing as previously reported.^58^ Both vectors silenced the endogenous WT human *HTT* with comparable efficiency after normalization for vector copy number (VCN) (Figure 2B). We observed a similar pattern in three independent experiments (chi-square test, degrees of freedom [df] = 47.54, 6; p < 0.0001).

### Efficiency of pCCL-shHTT6 in Striatal Neurons Derived from HD-iPSCs

**Figure 3:**
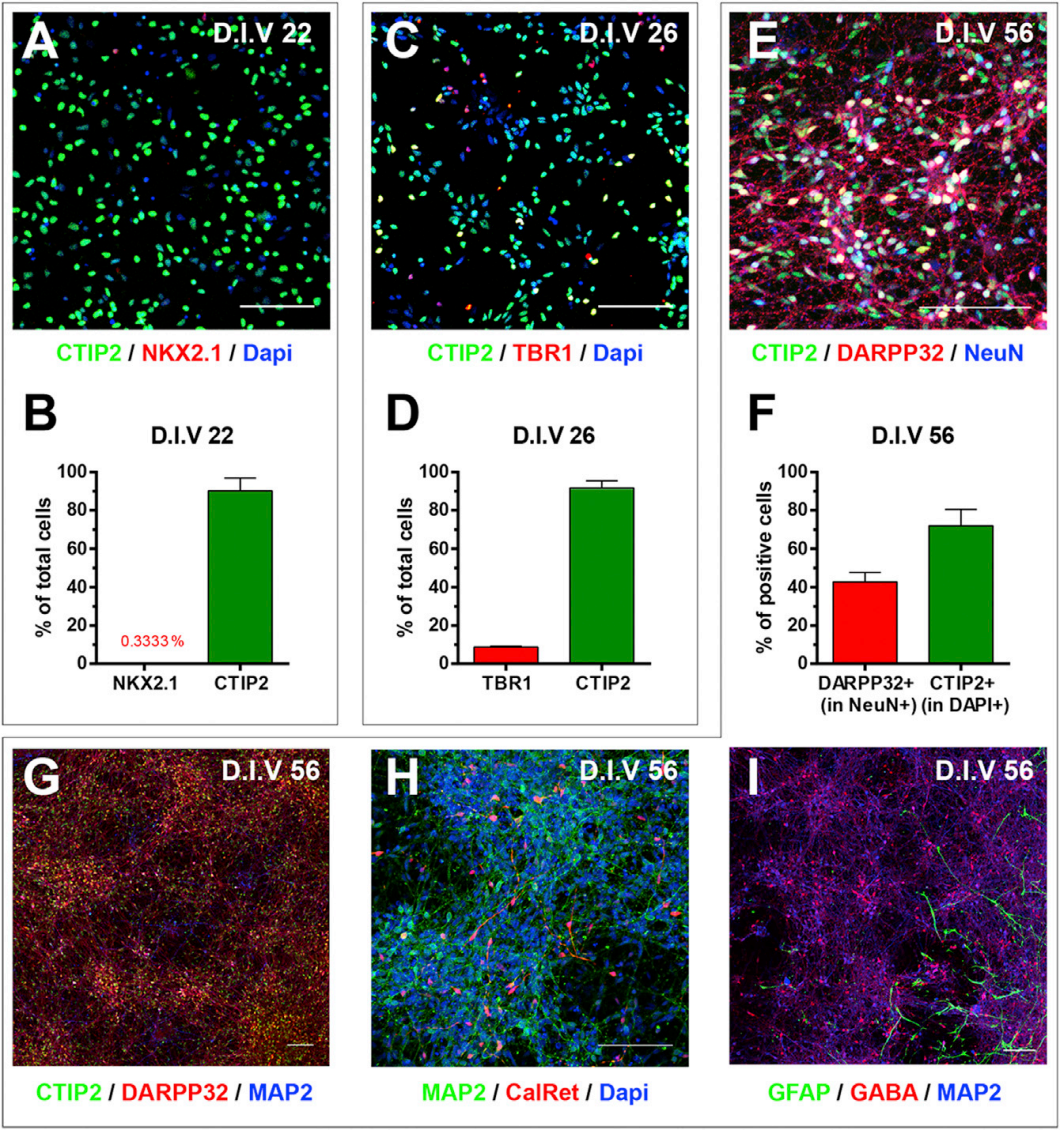
Striatal Identity of HD-iPSC-Derived Neural and Neuronal Derivatives (A) Immunolabeling at day 22 of NKX2.1 (red) and CTIP2 (green) in neuronal precursor cultures obtained from HD-iPSCs. (B) Quantification of NKX2.1- and CTIP2-expressing cells among all cells. (C) Immunolabeling at day 26 of TBR1 (red) and CTIP2 (green) in neuronal precursor cultures obtained from HD-iPSCs. (D) Quantification of TBR1- and CTIP2-expressing cells among all cells. (E) Immunolabeling at day 56 of DARPP32 (red), CTIP2 (green), and NeuN (blue) in neuronal cultures obtained from HD-iPSCs. (F) Quantification of DARPP32-expressing cells among NeuN-positive neurons and of CTIP2-expressing cells among all cells. (G–I) Immunolabeling at day 56 (21+35) of (G) DARPP32 (red), CTIP2 (green), and MAP2 (blue); (H) Calretinin (red) and MAP2 (green); and (I) GABA (red), GFAP (green), and MAP2 (blue) in neuronal cultures obtained from HD-iPSCs. Scale bars, 100 μm. Data are presented as the mean ± SEM. n = 3.

We further evaluated the activity of the pCCL-shHTT6 LV using human cultures enriched for medium spiny striatal neurons, derived from HD-iPSCs, as a relevant in vitro experimental paradigm.^59^ Human striatal neuronal cultures with the HD mutation were produced using an iPSC line derived from a patient carrying a 60 CAG triplet expansion in one allele of the *HTT* gene.^60^, ^61^ We first assessed the telencephalic dorso-ventral identity of the neuronal precursor cells after 22 and 26 days in vitro (DIV22/26) by immunofluorescence staining for CTIP2, NKX2.1, and TBR1 (Figures 3A–3D). At DIV22, 90.2% ± 3.8% of cells were CTIP2^+^, an early marker of medium spiny neurons^62^ (MSNs), whereas less than 0.3% ± 0.1% of cells expressed NKX2.1, a transcription factor necessary for MGE development that specifies the fate of non-striatal interneurons^62^, ^63^, ^64^ (Figures 3A and 3B). Similarly, at DIV26, 91.7% ± 3.7% of cells were CTIP2^+^, whereas only 8.7% ± 0.5% of cells expressed TBR1, a T-box transcription factor (TF) that plays a critical role in regulating the differentiation and identity of deep-layer projection neurons in the developing neocortex^65^, ^66^, ^67^, ^68^, ^69^ (Figures 3C and 3D). At DIV56, 72% ± 8.6% of cells still expressed CTIP2 and 42.7% ± 4.9% of NeuN^+^ neuronal cells co-expressed DARPP32 (PPP1R1B), an MSN-specific marker found co-expressed with CTIP2, but not by other cell types within the adult striatum^70^, ^71^, ^72^ (Figures 3E and 3F). Neuronal cultures enriched in MSNs (CTIP2^+^/DARPP32^+^/MAP2^+^) (Figure 3G) included other GABA^+^ neurons, such as Calretinin^+^ interneurons (Figure 3H), as well as a small number of GFAP^+^ astroglial cells (Figure 3I).

**Figure 4:**
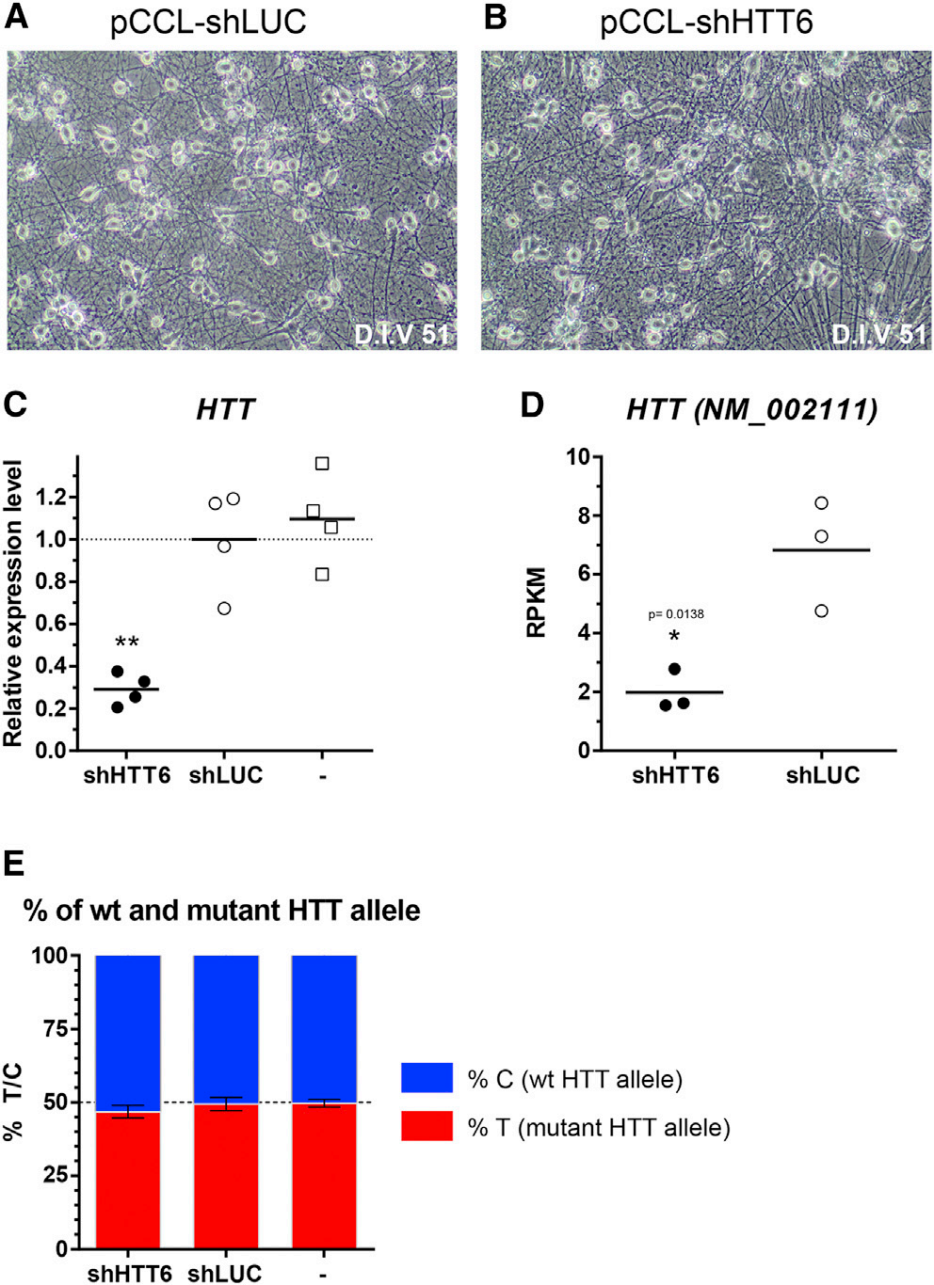
Quantification of HTT Knockdown by pCCL-shHTT6 Lentivirus in Human Striatal Neurons Derived from HD-iPSCs (A and B) Representative phase-contrast image of striatal neuronal culture derived from HD-iPSCs at day 51 (15 days after transduction with pCCL-shLUC [A] or pCCL-shHTT6 [B] lentivirus). (C) Relative *HTT* mRNA expression in striatal neuronal cultures transduced or not (−) with pCCL-shHTT6 and pCCL-shLUC. Expression levels are normalized to the mean levels detected in pCCL-shLUC-transduced cells. Values were compared for all groups by one-way ANOVA, and Dunnett’s multiple-comparison test was used to determine the level of significance. (D) RNA-seq expression data for HTT (NM_002111) expressed in reads per kilobase of transcript per million mapped reads (RPKM). Values for both groups were compared using the unpaired Student’s t test. (E) HTT allelic ratio expressed as the percentage of C or T allele at SNP rs362331. *p < 0.05; **p < 0.01.

These HD human striatal cells were infected at DIV36 with either pCCL-shHTT6 or pCCL-shLUC (Figures 4A and 4B) as a control. We have previously shown that under these experimental conditions the transduction efficiency is >85% (unpublished data). The corresponding mRNAs were extracted 15 days after transduction (at DIV51) and analyzed by qRT-PCR and next generation sequencing. qRT-PCR analyses show statistically significant knockdown of *HTT* gene expression (71% ± 7% [SD]; p < 0.01; Figure 4C). This result was confirmed by RNA sequencing (RNA-seq) data showing a similar knockdown effect of shHTT6 (71%; p = 0.0138; Figure 4D) on *HTT* transcript NM_002111. TaqMan qRT-PCR, based on SNP rs362331,^49^, ^73^ which is heterozygous in the HD-iPSC line, showed that the allelic ratio was not altered by shHTT6 treatment (Figure 4E).^74^ These results indicate that the pCCL-shHTT6 targets both the mutant and the WT *HTT* alleles in equal proportion. RNA-seq expression of classical neuronal markers revealed no significant effect of shHTT6 on the neuronal phenotype of the culture. The shHTT6/shLUC ratio remained close to 1.00 for *MAP2* (0.90), *TUBB3* (0.77), *SNAP25* (0.90), and *SYP* (0.92).

### Efficiency of pCCL-shHTT6 In Vivo

**Figure 5:**
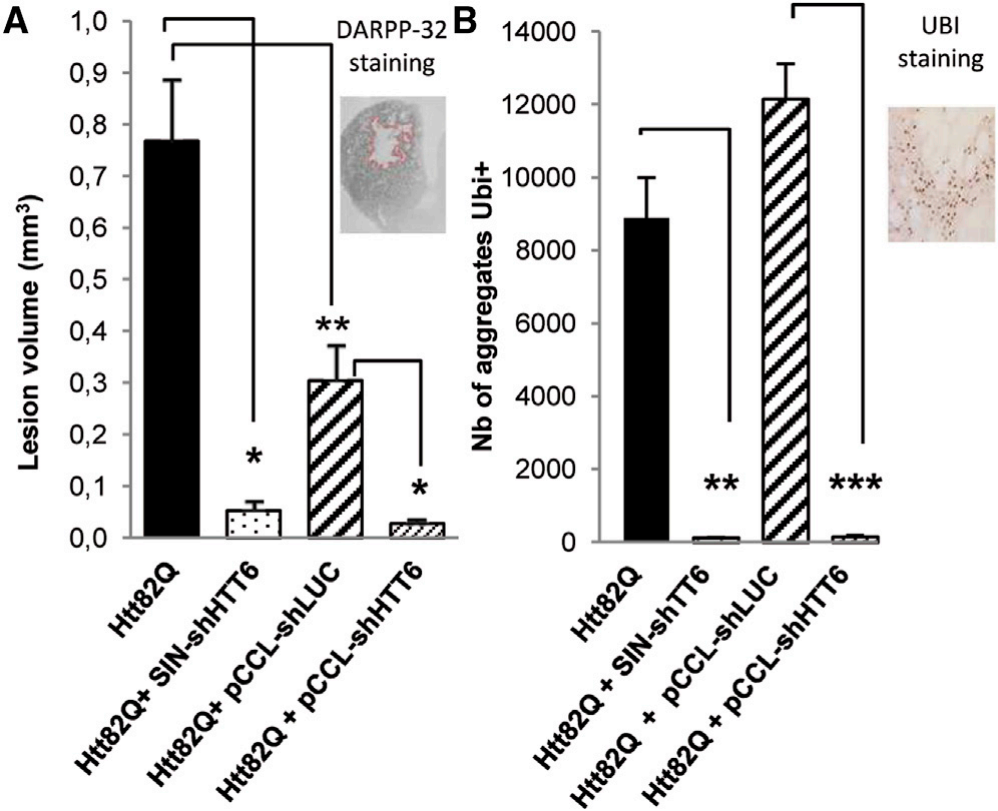
Evaluation of the Efficiency of HTT Silencing by SIN and pCCL-shHHT6 LVs In Vivo C57BL/6 mice were injected bilaterally in the striatum with 200 ng of LV encoding Htt171-82Q and shHTT6 or their respective negative control shRNA targeting luciferase. They were sacrificed 8 weeks later. (A) The size of the striatal lesion (expressed in mm^3^) was defined by the loss of DARPP-32 immunostaining, as illustrated in the insert. (B) The total number of ubiquitin-positive aggregates was counted in serial sections of the striatum, as illustrated in the insert. All values are expressed as the means ± SEM (n = 7–9). Groups were compared using the t test except for the comparison of lesion volume between Htt171-82Q and Htt171-82Q + SIN-shHTT6 for which the sign test was used (**p < 0.01; ***p < 0.001; ****p < 0.0001).

We injected the HTT171-82Q vector in the striatum of adult mice with either shHTT6 or shLUC in SIN or pCCL vectors to evaluate therapeutic efficiency in vivo. The severity of HD pathology was evaluated histologically 8 weeks after injection. Notably, striatal lesions, defined by the loss of DARPP-32 immunostaining, were almost completely eliminated by both pCCL-shHTT6 (t test, p < 0.01) and SIN-shHTT6 (sign test, p < 0.01): the size of the lesions was reduced by approximately 90% relative to the respective controls. The slightly lower loss of DARPP32 immunostaining in the control group (pCCL-shLUC) compared with the HTT171-82Q group is probably due to differences in the total dose of vector injected (400 ng versus 200 ng of p24, respectively), which might have affected the kinetic of the pathology. However, the number of ubiquitin (Ubi) aggregates, used to follow misfolded HTT (correlation demonstrated in Drouet et al.^49^), was similar in both groups. Finally, the number of HTT aggregates was equally reduced by both SIN (t test, p < 0.001) and pCCL-shHTT6 (t test, p < 0.0001) LVs (Figure 5). These results demonstrate the robust efficiency of pCCL-shHTT6 in vivo.

### Evaluation of the Biosafety of pCCL-H1-shHTT6

A major preclinical requisite is the demonstration of the biosafety of the vector. We investigated several parameters that could lead to potential side effects: (1) inappropriate synthesis and processing of the shRNA, (2) preferential incorporation of the passenger versus guide strand in the RNA-induced silencing complex (RISC), (3) off-target effects caused by partial homology of the guide RNA with other transcripts or pairing between the hexamer seed region and the 3′ UTR of transcripts (miRNA-like effects), (4) saturation of the endogenous cellular machinery, and (5) inflammatory/immune responses.

#### Strand Bias and Synthesis and Processing of shHTT6 In Vitro

**Figure 6:**
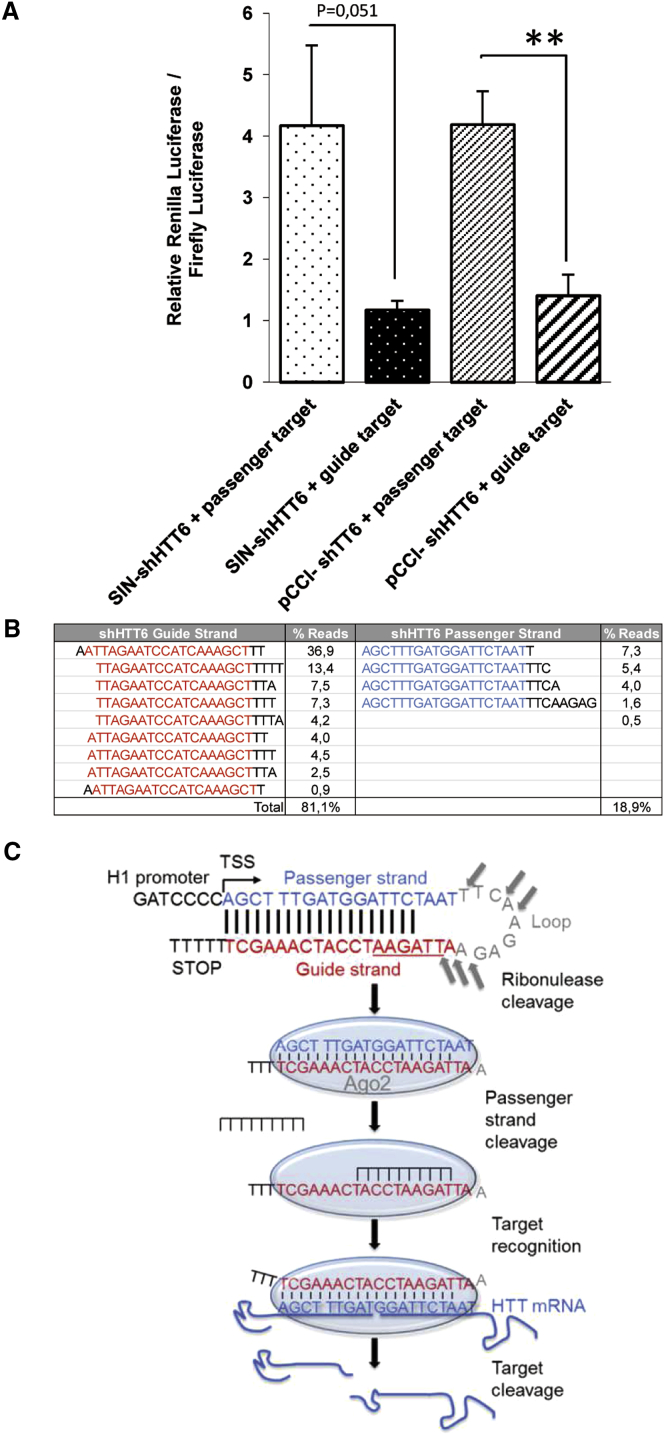
Evaluation of Strand Bias and shHTT6 Processing In Vitro (A) 293T cells were co-transfected with siRNA expression plasmids and a luciferase reporter plasmid containing either their intended guide target sequence or their unintended passenger target sequence. A DualGlo luciferase assay was performed 48 hr later. All values are expressed as the mean ± SEM (n = 4 for each group). Results show preferential guide strand incorporation in the silencing machinery for both SIN-shHTT6 (Mann-Whitney *U* test, p = 0.051) and pCCL-shHTT6 (t test, p < 0.01). (B) Distribution and percentage of small RNAs extracted from 293T cells infected with pCCL-shHTT6. Small RNAs were sequenced and aligned against the shHTT6 sequence. (C) Schematic representation of shHTT6 processing. The shRNA is transcribed in the nucleus of the infected cell and exported to the cytoplasm. Its loop is subsequently processed by a ribonuclease, generating a double-stranded RNA. The guide antisense strand is then loaded onto the Argonaute (Ago2) protein, a component of the RISC, whereas the passenger strand is cleaved and ejected. HTT target mRNA associates with the guide strand in RISC, is cleaved, and is subsequently degraded by cellular exonucleases. **p < 0.01.

We first investigated the incorporation of the guide and passenger strand in the RISC by the cellular machinery. Loading of the guide strand of the shRNA is essential to maximize silencing activity and minimize potential off-target effects. We used the psiCHECK-2 vector to monitor the incorporation of guide and passenger strands.^75^, ^76^ The target sequences of the guide and passenger strands of the shHTT6 were cloned downstream of the *renilla* luciferase translational stop codon. Target recognition and cleavage induce the degradation of *renilla* luciferase-HTT fusion mRNA. Measurement of the ratio between firefly and *renilla* luciferase activity is a convenient indicator of shHTT6 guide and passenger strand activity. In this experiment, the psiCHECK-2 reporter genes and pCCL-H1-shHTT6 were co-transfected into HEK293T cells. Consistent with the silencing data described above, pCCL-shHTT6 (t test, p < 0.01) and SIN-shHTT6 (Mann-Whitney *U* test, p = 0.051) efficiently reduced luciferase expression with preferential loading of the guide strand (Figure 6A).

We extracted and sequenced small RNAs from infected HEK293T cells to further analyze the processing of the shHTT6 guide strand. Between 100,000 and 270,000 reads per sample (n = 3) were aligned against the reference shHTT6 sequence (Figures 6B and 6C). The distribution and percentage of aligned reads showed that 81% corresponded to the guide strand of the shHTT6, confirming the psiCHECK-2 data. We observed cleavage of the loop in the aligned reads, in agreement with Dallas et al.,^77^ who showed that R-shRNAs have a cleavable loop that generates a 5′-phosphate at the 5′ end of the guide strand and that cleavage is necessary for optimum activity.

#### Identification of Possible shHTT6 Off-Targets

**Figure 7:**
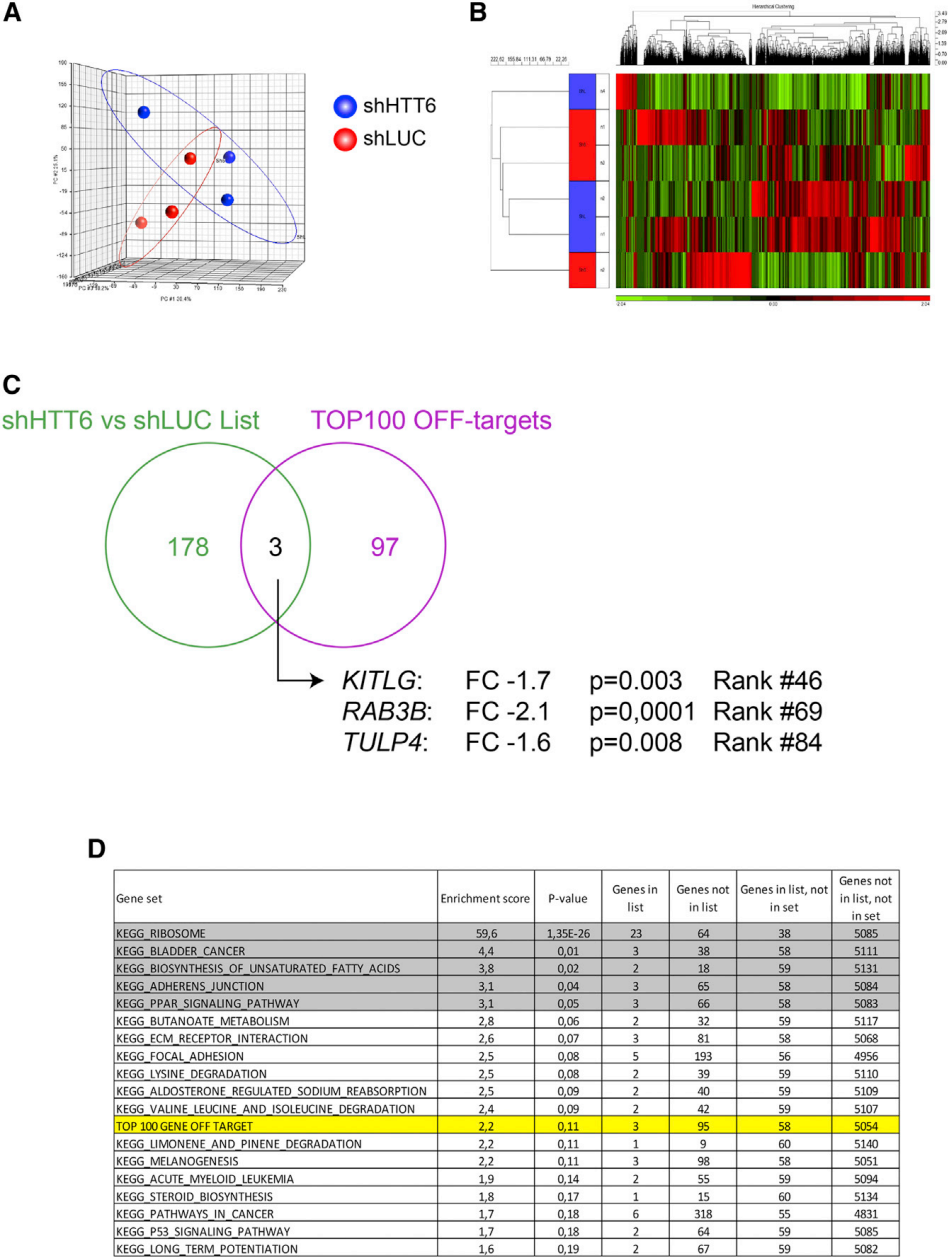
RNA-Seq Transcriptomic Analyses of pCCL-shHTT6 in HD-iPSC-Derived Human Striatal Neurons (A) PCA plot of PC1 versus PC2 versus PC3 of the transcriptomic profile of HD-iPSC-derived striatal neurons transduced with pCCL-shHTT6 or shLUC. (B) Hierarchical clustering and heatmap analysis of RNA-seq data. (C) Venn diagram depicting a non-significant overlap between the list of differentially expressed genes in shHTT6-treated human striatal neurons and the top 100 genes of the off-target list genes of shHTT6 (p = 0.05, Fisher exact test). (D) Top 20 most enriched from the KEGG pathway enrichment list (with TOP100 off-target list) in shHTT6-treated neurons.

Several algorithms have been developed to design small interfering RNAs (siRNAs) and analyze their potential off-targets.^78^, ^79^, ^80^, ^81^, ^82^ For shHTT6, we first searched for transcripts (exon, BLAST) with partial complementarity with the two strands and identified 15 and 5 human sequences with two to four mismatches with the guide and passenger strands, respectively (Table 1). In parallel, we performed an RNA-seq analysis of mRNAs extracted from human striatal cultures derived from HD-iPSCs at DIV51 transduced with pCCL-H1-shHTT6 or shLUC lentivirus at DIV36. We identified 181 differentially expressed genes in shHTT6-treated cells (fold change > 1.5; p < 0.01; Table S1). Levels for none of the theoretical off-target transcripts mentioned above were significantly altered following pCCL-H1-shHTT6 treatment. Furthermore, we used the siSPOTR software to identify a list of potential targets of the seed sequence of shHTT6 in the 3′ UTR of genes (“miR-like effect”) (Table S2). Again, the top 100 genes obtained with siSPOTR were crossed with RNA-seq data from human striatal cultures derived from HD-iPSCs treated with pCCL-H1-shHTT6 or shLUC. Principal component analysis and heatmaps (Figures 7A and 7B) of all expressed genes showed that shHTT6 treatment accounted for 10.44% of the total variance of transcriptomic profiles of the samples (with shHTT6 samples distinguished from shLUC samples along the third principal component axis [PCA]). Only three genes were present in both lists (KITLG, RAB3B, and TULP4; Figure 7C), revealing a non-significant overlap between the list of differentially expressed genes in shHTT6-treated human striatal neurons and potential off-targets (p = 0.05, Fisher exact test). Finally, we performed gene set enrichment analysis using the KEGG pathway enrichment list on our RNA-seq data (Figure 7D). The KEGG database (http://www.genome.jp/kegg/pathway.html) provides a collection of manually drawn pathways on molecular interactions and networks for biological interpretation and mapping of transcriptomic datasets. Only five KEGG gene sets were significantly enriched (Enrichment score > 1.5; p < 0.05) in shHTT6-treated samples. We then entered our theoretical TOP100 off-target list as a distinct gene set to determine whether it matched our RNA-seq data. The TOP100 off-target gene set ranked only 12^th^ and was not significantly enriched in our samples (embryonic stem [ES] = 2.2; p = 0.11) (Figure 7D). Altogether, the data demonstrate the excellent overall safety profile of pCCL-shHTT6.

**Table 1:** List of Transcripts with a Partial Homology with the Guide and Passenger Strands of shHTT6

Sequence (5′-3′)	Mismatches	Position	Name	Reference
**Transcripts with Partial Homology with shHTT6 Guide Strand5′-AGCT TTGATGGATTCTAAT-3′ HTT mRNA**				

AGTT TTCATGGATTCTAAT	2	exon 2	*Homo sapiens* chromosome 10 open reading frame 61 (C10orf61)	NM_015631.1 and AK128834
AGCT GAGATGGATTCTATT	3	exon 2	*Homo sapiens* DEAH (Asp-Glu-Ala-His) box polypeptide 8 (DHX8), mRNA	NM_004941.1 and BC038223
TGCT ATGATGAATTCTATT	4	exon-exon junction	*Homo sapiens* BRCA1 interacting protein C-terminal helicase 1 (BRIP1), mRNA	NM_032043.1 and AF360549
GGCT TTCATAGATTCTATT	4	exon 1	*Homo sapiens* angiopoietin like 2 (ANGPTL2), mRNA	NM_012098.2
GGCT TTAATGAATTCTCAT	4	exon 2	*Homo sapiens* dual adaptor of phosphotyrosine and 3-phosphoinositides (DAPP1), mRNA	NM_014395.1 and AF186022
GGCT TTGCTGGATTCTGCT	4	exon 2	*Homo sapiens* zinc finger protein 555 (ZNF555), mRNA	NM_152791.3 and AL832140
GGCT TTGCTGGATTCTGCT	4	exon 2	*Homo sapiens* zinc finger protein 57 (ZNF57), mRNA	NM_173480.1 and BX537601
GGCT TTGGTGGATTCTCAC	4	exon 1	*Homo sapiens* sema domain, immunoglobulin domain (Ig), transmembrane domain (TM) and short cytoplasmic domain, (semaphorin) 4F (SEMA4F), mRNA	NM_004263.2
GCCT TTTATGGCTTCTAAT	4	exon 1	cDNA FLJ37369 fis, clone BRAMY2024545	AK094688
AGCT TATAAGGATTCTAAT	3	EST	UI-CF-FN0-afo-j-12-0-Ul.s1 UI-CF-FNO *Homo sapiens* cDNA clone UI-CF-FNO-afo-j-12-0-UI 3-, mRNA sequence	CA314011
TGCT TTGAGGGCTTCTAAT	3	exon 1	*Homo sapiens* cDNA FLJ90150 fis, clone HEMBB1002039	AK074631
AGGA TTGATGGATTTTAAT	3	exon 1	full-length cDNA clone CS0DL008YM01 of B cells	CR616012.1

**Transcripts with Partial Homology with shHTT6 Passenger Strand5′-ATTAGAATCCATCAAAGCT-3′ HTT Antisense Sequence**				

ATAAGAATCGATCAAAGAT	3	exon 1	predicted: *Homo sapiens* KIAA0947 protein (KIAA0947), mRNA	XM_029101.9
ATTAGAACCCATCTAAACT	3	exon 1	*Homo sapiens* CDNAFLJ30271 fis, clone BRACE2002676	AK054833
AAAAGAATCCTTCAAAACT	4	exon 1	*Homo sapiens* ASAP (FLJ21159), mRNA	NM_024826.1
AGTAGAATCCATCATAGAA	4	exon 1	*Homo sapiens* multiple myeloma susceptibility mRNA sequence	AY094612
ACTAGTATGCATCAAAGCC	4	exon 1	*Homo sapiens* SET domain, bifurcated 1 (SETDB1), mRNA	NM_012432.2

The underlined text indicated the position of mismatched between the shHTT6 and the corresponding human genes.

#### In Vivo Evaluation of the Biosafety of Lentiviral-Delivered shHTT6

Previous studies have reported serious toxic and even lethal effects^54^, ^83^ caused by saturation of the endogenous silencing machinery when the shRNA is under the control of a strong promoter (in particular, the U6 promoter). Liver failure and mortality were observed in mice injected with different adeno-associated virus (AAV)-expressed shRNAs, irrespective of hairpin length, sequence, or target transcript. However, this cytotoxicity is relieved by the use of the weaker H1 promoter.^84^ We therefore assessed the gene expression profile of representative genes of the cellular miRNA machinery using custom PCR arrays. Exportin-5 and Dicer-1 expression were not significantly altered at the effective dose of 200 ng, up to 6 weeks after injection (Table 2).

**Table 2:** Evaluation of Expression Changes of Inflammatory Genes after Injection of LVs

				24 hr Post-injection							6 Weeks Post-injection					
				100 ng		200 ng		Non-purified 200 ng		Positive Control 1,000 ng	100 ng		200 ng		Non-purified 200 ng	
UniGene	RefSeq	Symbol	Description	p Value	Fold Change	p Value	Fold Change	p Value	Fold Change	Fold Change	p Value	Fold Change	p Value	Fold Change	p Value	Fold Change
**RNA/DNA Sensors**																

Mm.33874	NM_126166.4	TLR3	toll-like receptor 3	0.1400	1.7	0.0770	2.4	0.1787	1.3	103.6	0.3996	1.20	0.0867	2.8	0.0743	2.0
Mm.23979	NM_133211.3	TLR7	toll-like receptor 7	0.3651	1.1	0.4514	−1.1	0.0170^a^	−1.8	112.0	0.4413	1.12	0.0874	2.9	0.0698	2.1
Mm. 196676	NM_133212.2	TLR8	toll-like receptor 8	0.3642	1.1	0.4571	−1.1	0.0121^a^	−2.0	112.0	0.4413	1.12	0.0874	2.9	0.0698	2.1
Mm.44889	NM_031178	TLR9	toll-like receptor 9	0.1063	1.8	0.0720	2.6	0.0066^a^^,^^b^	2.0^b^	102.8	0.4413	1.12	0.0874	2.9	0.0698	2.1
Mm.277250	NM_011871.2	PKR	protein kinase, interferon-inducible double-stranded RNA-dependent activator	0.8388	−1.0	0.5392	1.3	0.1463	−1.7	24.2	0.1151	2.36	0.6986	1.5	0.1891	2.1
Mm.296366	NM_009021.2	RIG-1	retinoic-acid-induced 1 transcript variant 1	0.7755	1.0	0.5436	1.1	0.6895	−1.2	71.4	0.3822	1.27	0.0750	2.7	0.2040	1.8
Mm.136224	NM_001164477.1	Ifihl	interferon induced with helicase C domain 1 (MDA-5)	0.0883	1.9	0.0328^a^^,^^b^	3.6^b^	0.0040^a^^,^^b^	3.0^b^	112.0	0.4413	1.12	0.0874	2.9	0.0698	2.1
Mm.222633	NM_145857.2	Nod2	nucleotide-binding oligomerization domain containing 2	0.1263	1.5	0.1076	1.9	0.0210^a^^,^^b^	2.0^b^	73.7	0.4061	1.13	0.0635	3.0	0.0873	1.8

**Downstream Effectors of Immune Response**																

Mm.213003	NM_010851.2	Myd88	myeloid differentiation primary response gene 88	0.3651	1.1	0.4092	1.0	0.4769	−1.3	112.0	0.4413	1.12	0.0874	2.9	0.0698	2.1
Mm.149280	NM_173394.2	Ticam2	toll-like receptor adaptor molecule 2 (TRIF)	0.3682	1.1	0.4568	−1.1	0.0113^a^^,^^b^	−2.1	112.0	0.4413	1.12	0.0874	2.9	0.0698	2.1
Mm.3960	NM_016849.3	IRF-3	interferon regulatory factor 3	0.3714	1.1	0.4037	1.1	0.0375^a^	−1.7	110.9	0.4413	1.12	0.0874	2.9	0.0698	2.1
Mm.3233	NM_016850.2	IRF-7	interferon regulatory factor 7	0.0079^a^^,^^b^	3.2	0.0505	7.6	0.0007^a^^,^^b^	3.7	9.4	0.3365	1.32	0.0404^a^	4.1	0.0613	2.2
Mm.322843	NM_020583.5	ISG20	interferon-stimulated protein (Isg20), transcript variant 1	0.0174^a^^,^^b^	4.6	0.0082^a^^,^^b^	10.9	0.0001^a^^,^^b^	8.6	14.9	0.3468	1.28	0.0551	3.4	0.0441^a^^,^^b^	2.4^b^
Mm.256765	NM_008689.2	NF-κB1	nuclear factor kappa-B, subunit 1	0.9617	1.0	0.2132	1.5	0.0642	1.8	22.8	0.1628	1.36	0.0920	2.3	0.0907	1.6
Mm.292547	NM_011198.3	PTSG	prostaglandin-endoperoxide synthase 2	0.5842	1.2	0.3723	1.4	0.1611	1.6	60.3	0.4271	1.14	0.0892	2.8	0.0711	2.0
Mm.277406	NM_009283.3	STAT-1	signal transducer and activator of transcription 1	0.0031^a^^,^^b^	3.0^b^	0.0001^a^^,^^b^	5.0	0.0005^a^^,^^b^	3.2	1.7	0.4792	1.24	0.0508	3.1	0.1650	1.4
Mm.293120	NM_019963.1	STAT-2	signal transducer and activator of transcription 2	0.1185	3.3	0.0099^a^^,^^b^	4.5	0.0110^a^^,^^b^	2.8	2.3	0.0622	1.89	0.5128	1.2	0.1167	1.5
Mm.95479	NM_145209.3	2′5′OAS	2′-5′ oligoadenylate synthetase-like 1	0.0440^a^	1.8	0.0178^a^^,^^b^	3.1	0.0022^a^^,^^b^	4.0	92.7	0.4413	1.12	0.0874	2.9	0.0698	2.1
Mm.439751	NM_008331.3	IFIT-1	interferon-induced protein with tetratricopeptide repeats 1	0.0602	4.2	0.0022^a^^,^^b^	10.5	0.0001^a^^,^^b^	8.8	4.9	0.3076	1.33	0.0337^a^	4.1	0.0402^a^	2.3
Mm.2036	NM_008332.3	IFIT-2	interferon-induced protein with tetratricopeptide repeats 2	0.0421^a^^,^^b^	8.6^b^	0.1411	17.8	0.0001^a^^,^^b^	11.7	48.6	0.1747	1.49	0.0156^a^	3.3	0.1138	1.8
Mm.19131	NM_009778	C3	complement component 3	0.2833	1.1	0.1758	2.1	0.0060^a^^,^^b^	3.1	20.7	0.3720	1.25	0.0629	3.4	0.0482^a^	2.3
Mm.24045	NM_021384.3	CIG-5	radical S-adenosyl methionine domain containing 2	0.0473^a^^,^^b^	4.6^b^	0.0879	16.1	0.0001^a^^,^^b^	12.4	25.6	0.2050	1.41	0.0754	4.8	0.2567	3.3

**Cytokines**																

Mm.222830	NM_008361	II lb	interleukin-1 beta	0.2980	1.6	0.1648	1.9	0.0006^a^^,^^b^	4.8	12.6	0.4413	1.12	0.0874	2.9	0.0698	2.1
Mm.1019	NM_031168.1	IL-6	interleukin-6	0.2141	1.5	0.1873	1.7	0.0088^a^^,^^b^	2.3	112.0	0.4413	1.12	0.0874	2.9	0.0698	2.1
Mm.1245	NM_010510.1	Ifnbl	interferon-beta 1	0.2563	1.3	0.1017	2.5	0.5754	1.1	112.0	0.4413	1.12	0.0874	2.9	0.0698	2.1
Mm.240327	NM_008337	Ifng	interferon-gamma	0.3682	1.1	0.4068	1.1	0.1111	−1.5	112.0	0.4413	1.12	0.0874	2.9	0.0698	2.1
Mm.1293	NM_013693	Tnf	tumor necrosis factor DIF/TNF-alpha	0.1380	1.6	0.0775	2.5	0.0022^a^^,^^b^	2.1^b^	101.3	0.4413	1.12	0.0874	2.9	0.0698	2.1

**Chemokines**																

Mm.290320	NM_011333	Ccl2	chemokine (C-C motif) ligand 2	0.2305	2.4	0.2039	7.2	0.0023^a^^,^^b^	5.4^b^	9.7	0.4413	1.12	0.0874	2.9	0.0698	2.1
Mm.1282	NM_011337	Ccl3	chemokine (C-C motif) ligand 3	0.8226	1.1	0.5772	2.0	0.1268	2.2	17.1	0.4413	1.12	0.0874	2.9	0.0698	2.1
Mm.244263	NM_013652	Cd4	chemokine (C-C motif) ligand 4	0.0333^a^^,^^b^	2.8^b^	0.1477	6.4	0.0015^a^^,^^b^	4.7^b^	74.9	0.4413	1.12	0.0874	2.9	0.0698	2.1
Mm.284248	NM_013653	Ccl5	chemokine (C-C motif) ligand 5 MuRantes/RANTES	0.9804	1.3	0.1071	2.4	0.8379	1.5	1.6	0.4413	1.12	0.0519	3.4	0.0650	2.2
Mm.877	NM_021274	Cxcl10	chemokine (C-X-C motif) ligand 10	0.2693	3.0	0.0324^a^	7.1^b^	0.0157^a^	3.3^b^	3.6	0.4232	1.15	0.0874	2.9	0.0685	2.1

**Markers of Cellular Immune Response**																

Mm.1858	NM_009857.1	Cd8a	CD8 antigen, alpha chain (isoform 2)	0.3467	1.2	0.4333	−1.0	0.1374	−2.0	112.0	0.4413	1.12	0.0874	2.9	0.0698	2.1
Mm.15819	NM_009853.1	Cd68	CD68 (macrosialin)	0.9278	−1.0	0.2318	1.4	0.7309	1.1	2.1	0.9799	1.03	0.1114	1.6	0.5726	1.1
Mm.210361	NM_007648.4	Cd3e	CD3 antigen, epsilon polypeptide	0.3408	1.2	0.4428	−1.1	0.0306^a^	−1.7	112.0	0.4413	1.12	0.0874	2.9	0.0698	2.1
Mm.2209	NM_013488	Cd4	CD4 antigen	0.0413^a^	−1.5	0.2243	−1.5	0.1450	−1.3	7.6	0.8866	1.02	0.3322	1.3	0.4661	−1.5
Mm.10747	NM_019467.2	Aif1	allograft inflammatory factor 1 (ibal)	0.7214	1.0	0.7278	−1.1	0.9628	1.3	4.7	0.0147^a^	1.76	0.0625	2.4	0.0365^a^	1.6
Mm.262106	NM_008401	Itgam	integrin alpha M (CD11b)	0.1692	−1.5	0.1552	−1.5	0.2742	−1.3	4.1	0.0236^a^	1.65	0.0242^a^^,^^b^	2.2^b^	0.1886	1.6
Mm.2254	NM_010130.4	Emr1	EGF-like module-containing, mucin-like, hormone receptor-like sequence 1 precursor (F4/80)	0.0960	−2.2	0.1020	−1.9	0.0788	−2.2	5.5	0.1388	1.49	0.0045^a^^,^^b^	4.0^b^	0.0164^a^^,^^b^	2.0
Mm.1239	NM_010277	Gfap	glial fibrillary acidic protein	0.1444	−1.2	0.6905	−1.2	0.8128	1.0	2.0	0.8252	−1.07	0.0829	2.6	0.7093	−1.1

**Silencing Machinery**																

Mm.21135	NM_148948.2	Dicerl	Dcr-1 homolog (dicerl)	0.3626	1.6	0.3342	1.6	0.3760	1.5	39.2	0.0471^a^^,^^b^	2.99^b^	0.2055	2.1	0.2758	2.0
Mm.275039	NM_028198.2	Xpo5	exportin5	0.4338	−1.1	0.5410	1.1	0.8085	−1.0	6.2	0.1200	1.42	0.0816	1.9	0.2471	1.4

**Housekeeping Genes**																

Mm.2180	NM_008302	Hsp90ab1	heat shock protein 90 alpha (cytosolic), class B member 1	0.8615	1.0	0.3805	−1.3	0.5394	−1.1	−1.5	0.8154	−1.03	0.2961	−1.7	0.4889	1.1
Mm.343110	NM_008084	Gapdh	glyceraldehyde-3-phosphate dehydrogenase	0.7310	−1.0	0.3694	1.3	0.6718	1.1	1.5	0.7376	1.03	0.1746	1.7	0.7591	−1.1

C57BL/6 mice were injected bilaterally in the striatum with 100 or 200 ng of purified LV encoding pCCL-shHTT6, 200 ng of non-purified LV, or vehicle PBS as a negative control. They were sacrificed 24 hr or 6 weeks later to evaluate the presence of an immune reaction in the brain. A positive control mouse injected with a high dose of LV (1,000 ng, LV = SIN-shHTT6) was also sacrificed at 24 hr. The profile of inflammatory gene expression was determined using custom PCR arrays on striatum punches from both injection sites, with values normalized to the average of the expression of housekeeping genes. Presented values are the fold change of expression relative to the PBS-injected group.

a Genes showing a statistically significant (t test) change less than 2-fold.

b Genes showing an at least statistically significant (t test) ± 2-fold change.

Therapeutic efficacy should not be compromised by an innate or adaptive immune response following LV administration to the striatum. We used ion exchange purified LV batches to reduce the presence of contaminants that could potentially induce an inflammatory/immune response. Striatal samples were collected after injection of LVs at two doses (100 ng and 200 ng), either 24 hr later, to assess a representative response to the vector, or 6 weeks after injection, likely to reflect the response to the shHTT6 transgene.^85^, ^86^, ^87^ We added a group injected with 200 ng of non-purified LV to examine the relative contribution of contaminants produced during LV preparation (such as cell debris or serum) to the response, and a positive control sample from a mouse injected with a high dose of LV (1,000 ng, SIN-shHTT6) to establish the range of a strong immune response. We determined the profile of inflammatory gene expression using custom PCR arrays for intracellular receptors sensitive to double-stranded RNA, such as Toll-like receptors (TLRs) 3, 7, 8, 9, PKR, and RIG-1 and several of their downstream effectors. For example, it has been shown that the TLR3 response leads to interferon regulatory factor 3 (IRF-3) phosphorylation and nuclear factor κB (NF-κB) activation, triggering the expression of inflammatory cytokines, such as tumor necrosis factor (TNF) alpha, interleukin (IL)-6, and chemokines (CCL-5, CCL-10). These, in turn, stimulate interferon (IFN) beta and interferon-stimulated genes, such as 2′5′-oligoadenylate synthetase (OAS1).^88^ Similarly, activation of the protein kinase PKR induces multiple interferon response genes, such as OAS1.^89^ We also examined the expression of markers of cellular immune responses for both the systemic system and CNS. Our study shows that striatal injection of research grade LV was associated with slight upregulation of several genes (namely MDA-5, a viral sensor, several downstream effectors of the immune response, such as ISG20, STAT1 and STAT2, OAS1, IFIT-1, and only one chemokine [Cxcl10]), 24 hr following surgery (Table 2). However, very few genes remained upregulated 6 weeks later, such as IRF-7, IFIT-1, and IFIT-2. Moderate overexpression of F4/80, a marker for macrophages, and CD11b, a marker for macrophages and microglia, appeared at this late time point. As expected, purification of the LV batch reduced the immune reaction associated with the injection, particularly shortly after injection in the brain. Altogether, our data suggest that an effective dose of purified shHTT6 LV is not associated with side effects in the brain.

## Discussion

Several novel therapeutic strategies are currently under development to downregulate the expression of *HTT* as a treatment for HD. Yet, a number of safety issues still have to be addressed to ensure long-term beneficial effects for the patient, while minimizing potential adverse events. Here, we optimized an LV encoding an shRNA targeting *HTT*, which was evaluated in the striatum of HD transgenic mice and neurons derived from HD iPSCs. We report not only a robust reduction of two major pathological hallmarks for HD, but also a minimal inflammatory response in the brain, proper cellular processing of the shRNA, and a highly favorable on/off-target profile.

A correlation has been observed between siRNA efficacy and transcript abundance or turnover, with low-abundance and short-lived mRNAs being less sensitive to siRNA treatment.^27^, ^28^, ^29^, ^30^, ^31^, ^32^ In the present study, we obtained similar levels of *HTT* silencing against exogenous human mutant *HTT* expressed at a very high level^17^ (HEK293T cells and adult mice) and endogenous WT and mutant *HTT* (HEK293T cells and HD-iPSCs), confirming the potency of 19-nt-long shRNAs (right and left types; R and L shRNAs).^90^ These shRNAs are processed by a DICER-independent pathway, which has also been reported for miR-451.^91^, ^92^, ^93^, ^94^, ^95^ This alternative Argonaute (Ago2)-mediated processing induces guide-specific RNAi activity.^96^ This was confirmed by sequencing, which showed preferential detection of the guide strand and the processing of shHTT6 with the expected cleavage of the loop.

Aside from silencing efficiency, the biosafety of various *HTT* silencing approaches is paramount for guiding clinical development. Here, we chose to design our siRNA into an LV encoding a short hairpin RNA to simplify delivery via a unique stereotaxic surgery in the brain of patients. Transduction of neurons with LVs ensures long-term and continuous expression of siRNAs in the affected brain region, in contrast with repeated administrations necessary for treatment with antisense nucleotides, peptides, nucleic acids, or siRNAs. However, low Exportin-5 levels or the number of active RISCs in the brain may increase susceptibility to adverse shRNA-induced saturation effects and neurotoxicity.^97^, ^98^, ^99^ Here, we confirm that the weak H1 polymerase III promoter does not induce saturation of the miRNA machinery (in particular, Exportin-5) or induce interferon response and downstream effectors.^51^, ^52^, ^53^

We further addressed the safety of pCCL-shHTT6 silencing by investigating potential recognition and degradation of mRNA with imperfect complementarity in the coding region and 3′ UTR of human genes. None of the 20 human exons with partial homology with the guide and passenger strand of shHTT6 were differentially expressed in differentiated HD-induced pluripotent stem (iPS) cultures. Cross-evaluation of the top 100 candidates with shHTT6 seed sequences and next generation sequencing (NGS) data from HD-iPSCs show limited overlap, demonstrating the specificity of shHTT6. Three genes were present in both lists: KITLG, RAB3B, and TULP4. They were not among the top differentially expressed genes (rank 46, 69, and 84) or among the most highly ranked off-target candidates. We cannot exclude that these gene expression changes are due to on-target HTT silencing and the subsequent biological effects or variability in the differentiation state or populations of various cultures. KEGG analysis of differentially expressed genes identified pathways that have been previously associated with HTT biology and revealed that the gene set of off-target candidates is not enriched in treated samples.^100^, ^101^, ^102^, ^103^ Altogether, the data demonstrate the excellent overall safety profile of pCCL-shHTT6.

Our in vivo data also showed a transient and modest increase in the expression of the viral sensor MDA-5 and several downstream effectors of the immune response 24 hr after surgery. Six weeks after injection, only a few markers of resident microglia and macrophages were still slightly elevated, with no obvious astrocyte activation. No alterations of CD4 or CD8 mRNA were detected, suggesting the absence of cell-mediated responses. We also observed no induction of major cytokine or chemokine expression in the brain, despite reports of interferon-mediated responses by others.^89^, ^104^, ^105^ Our data are in accordance with studies showing that long-term expression of shRNA, cloned in an miRNA backbone and vectorized in AAV, triggered similarly mild immune responses.^106^, ^107^

pCCL-shHTT6 induces global silencing of both mutant and WT HTT alleles. Thus, all HD patients could be treated with a single product. In contrast, allele-specific silencing, based on SNPs to discriminate between the mutant and WT allele, is limited to a subset of HD patients.^108^ However, a delicate balance should be established between downregulating mutant HTT sufficiently to alleviate disease pathology, while preserving enough WT HTT to maintain its physiological roles. Early *HTT* KO mouse models clearly demonstrated that HTT is important for embryonic development because these mice die at embryonic day 8.^18^, ^19^, ^20^ Nonetheless, inducible depletion of HTT in the adult brain (after 4 months of age) does not cause any cerebral atrophy, neurodegeneration, or motor defects.^21^ Similarly, two studies in adult non-human primates also reported that partial suppression (between 30% and 45%) of WT HTT expression is well tolerated from 6 weeks up to 6 months after treatment with AAV vectorized shRNAs.^106^, ^107^ More recently, studies using a CRISPR/Cas9 approach to inactivate mutant *HTT* expression have reported very encouraging results, but a neuropathological assessment of therapeutic efficacy is still awaiting.^109^, ^110^ Here, pCCL-shHTT6 downregulated human *HTT* expression in transduced HEK293T cells and striatal neurons derived from HD-iPSCs by approximately 60%–70%, leaving both remaining mutant and WT HTT. RNA-seq data also revealed that the recently described antisense HTT transcript (HTT-AS1)^111^ is barely detected in these human neurons (below detection level in five of six samples), and therefore does not interfere with the silencing of HTT by pCCL-shHTT6. In an HD mouse model, a single striatal injection with pCCL-shHTT6 LV was sufficient to eliminate the DARPP-32 lesion and almost abolish the formation of Ubi-positive aggregates for up to 6 weeks. Altogether, these data suggest that the level of HTT suppression induced by pCCL-shHTT6 is therapeutically efficient and should be safe in the long term. One challenge for the clinical development of our approach is the scale-up to humans. We have previously shown that LV transduces 1–1.5 × 10^6^ neurons (50–60 mm^3^) in the putamen of cynomolgus monkeys.^112^ However, the development of LVs with retrograde properties,^113^ new injection procedures,^114^ and connectome data^115^ provide new opportunities to target specific and highly relevant sub-regions of the brain to maximize the therapeutic benefit.

## Materials and Methods

### Plasmids

pCCL-hPGK-nls-LacZ was kindly provided by Prof. Luigi Naldini.^116^ The RRE-hPGK-nls-LacZ-3′ LTR was replaced with the mPGK-nls-LacZ-WPRE-SIN-3′ LTR fragment from SIN-mPGK-nls-LacZ-WPRE-SIN-3′ LTR^117^ to generate pCCL-mPGK-nls-LacZ-WPRE-SIN. The XbaI site after the 3′ LTR was removed to generate pCCL-mPGK-nls-LacZ-WPRE-SINΔXbaI. Finally, pCCL-H1-shHTT6 and pCCL-H1-shLUC were generated from pENTR/D-TOPO-H1-shHTT6 and pENTR/D-TOPO-H1-shLUC by digesting them with NotI blunt and PvuII and cloning the H1-shHTT6 and H1-shLUC fragments into ClaI-EcoRI blunt digested pCCL-mPGK-nls-LacZ-WPRE-SINΔXbaI. LVs encoding the first 171 amino acids of human HTT with 82 CAG repeats (SIN-PGK-HTT171-82Q-WPRE) and the shRNA that targets human HTT (SIN-cPPT-PGK-GFP-WPRE-LTR-TRE-H1-shHTT6, hereafter called SIN-shHTT6) have been previously described.^17^, ^118^

The psiCHECK-2 plasmid, expressing *renilla* and firefly luciferase (Promega), was used for rapid, quantitative evaluation of guide and passenger strand incorporation into the RISC.^75^, ^76^ The target sequences of shHTT6 (guide and passenger stands) were cloned into the multiple cloning region located downstream of the *Renilla* luciferase translational stop codon. The psiCHECK-2 plasmid was digested with XhoI and NotI and gel purified. Insert DNA containing the shHHT6 passenger (5′-TCGAGAAAGCTTTGATGGATTCTAATCTGGATCCGC-3′ and 5′-GGCCGCGGATCCAGATTAGAATCCATCAAAGCTTTC-3′) and guide strands (5′-TCGAGAGATTAGAATCCATCAAAGCTTTGGATCCGC-3′ and 5′-GGCCGCGGATCCAAAGCTTTGATGGATTCTAATCTC-3′) were hybridized (95°C, cooled to room temperature [RT]) and ligated with T4 ligase (New England Biolabs) into the digested psiCHECK-2 vector. The resulting plasmids, psiCheck-2-shHTT6/G and psiCheck-2-shHTT6/P, were verified by sequencing (sequencing primer: 5′-TCAAGAGCTTCGTGGAG-3′).

### LV Production and Purification

LVs were produced in HEK293T cells, using the four-plasmid system, as previously described.^119^ HIV-1 vectors were pseudotyped with the vesicular stomatitis virus glycoprotein (VSV-G) envelope, concentrated by ultracentrifugation, and resuspended in PBS (GIBCO, Life Technologies) supplemented with 1% BSA (Sigma-Aldrich).

The Vivapure LentiSELECT 500 kit was used for small-scale purification of the lentiviral particles (VWR). The Sartobind ion exchange membrane adsorber technology used in LentiSELECT efficiently and rapidly captures and recovers large viral particles (3,000 nm pores). In brief, 500 mL of supernatant was harvested and filtrated on the provided filter. The supernatant was pumped through the filter at a rate of 10–20 mL/min. After the assembly of the LentiSELECT column and connection to the peristaltic pump, 150 mL of loading buffer was pumped through the column at a rate of 10 mL/min. The flow through was treated as biohazard waste. Once bound, viral particles were purified by washing away nonspecifically bound protein with 120 mL of washing buffer at a rate of 15 mL/min. The viral particles were then eluted using 30 mL of buffered solution containing a high concentration of sodium chloride. The elution was performed using a 20 mL syringe at a rate of 1 mL/min. Finally, the eluate was collected in PBS and centrifuged at 37,000 × *g* at 4°C for 90 min to concentrate it. The viral pellet was resuspended in PBS, aliquoted, and frozen at −80°C.

The viral particle content of each batch was determined by p24 antigen ELISA (RETROtek). Viral stocks were stored at −80°C until use.

### HEK293T Cell Culture, Infection, RNA Extraction, and qRT-PCR

HEK293T cells (CRL11268; ATCC/LGC standards) were cultured in DMEM supplemented with 10% fetal bovine serum (FBS), 100 U/mL penicillin, and 100 μg/mL streptomycin at 37°C in a 5% CO2/air atmosphere. One day prior to infection, HEK293T cells were plated at a density of 500,000 cells per well in 6-well plates (Becton Dickinson) or 100,000 cells per well in 12-well plates (Becton Dickinson). One day later, the cells in the 6-well plates were co-infected with lentiviral particles (150 ng of p24 antigen for each LV) expressing Htt171-82Q, and the shRNAs and the cells in the 12-well plates with 60 ng of p24 with the pCCL-shHTT6 per well. They were passaged once 48 hr later, and TRIzol RNA Isolation Reagent (Thermo Fisher) was used to isolate the mRNA for qRT-PCR analysis 5 days after infection. For the analysis of shRNA synthesis and processing, cells from each 12-well plate were passaged and transferred into two 6-well plates, 2 days after infection, and the mirVana miRNA Isolation kit (Life Technologies) was used to extract total RNA 3 days later. For the experiment to measure VCN, HEK293T cells were infected with lentiviral particles (150 ng of p24 antigen) expressing the shRNAs (SIN-shHTT6 or shLUC and pCCL-shHTT6 or shLUC), and the corresponding DNA and RNA (six wells; Corning Life Sciences) were extracted with the TRIzol RNA Isolation Reagent (Thermo Fisher), 7 days after infection. All samples were stored at −80°C.

### Luciferase Assays

Five thousand HEK293T cells were co-transfected with 0.7 μL of TransFast transfection buffer (E2431; Promega), 60 ng of shHHT6 plasmids, and 20 ng of psiCHECK-2 or psiCHECK-2 containing the HTT6 guide/passenger sequence. Transfected cells were assayed 48 hr later, according to the manufacturer’s protocol for the Luciferase Reporter Assay System (Promega) using a Glomax 96 multiplate luminometer (Promega). Relative luciferase activity was calculated as the ratio between *renilla* and firefly luciferase activities × 100.

### Measurement of HTT Silencing and Vector Copy Number by Cross-Species Duplex qPCR

We used the recently developed and validated primers for assessment of LV copy in various species including humans and rodents. The primers for the determination of VCN were LV-HIV-F: 5′-TCTCGACGCAGGACTCG-3′ and LV-HIV-R: 5′-TACTGACGCTCTCGCACC-3′, targeting the LV,^120^ and the highly conserved Poly (rC)-binding protein 2 (PCBP2) gene PCBP2-F: 5′-TTGTGTCTCCAGTCTGCTTG-3′, PCBP2-R: 5′-AGGTGGTGGTGGTGGTA-3′.^121^

Following the workflow described by Christodoulou et al.,^58^ 100 ng of genomic DNA (gDNA) from HEK293T cells was used for the qPCR. For the standard curve, we used plasmid amounts (16 pg to 2 ng) of pCCL-shLUC equivalent to a VCN of 0.16 to 20 in 100 ng of gDNA, adjusting plasmid amounts for the size of the human genome (3.1 Gb per haploid genome). The qPCR was performed in duplicate in 20 μL using the KAPA SYBR FAST qPCR kit (Axon Laboratories) and 300 nM of each primer with a standard PCR program of 5 min at 95°C followed by 40 cycles of 3 s at 95°C, 20 s at 60°C (Rotor-Gene Q; QIAGEN). The qPCR duplex assay displayed average amplification efficiencies of 92.4% ± 3.1% for qLV and 89.4% ± 1.2% for qPCBP2, and a good correspondence between the expected and observed LV values was observed in all cases. We used the spreadsheets from Christodoulou and et al.^58^ to calculate the LV plasmid and gDNA standard curves. The VCN standard curves and quantification of LV insertion sites in samples were calculated using the ΔΔCT method values with PCBP2 as the internal calibrator gene. The analysis was performed on three independent experiments with biological duplicates or triplicates (total of n = 6). Technical duplicates were used for the qPCR and qRT-PCR analysis. Chi-square analysis was used to evaluate the reproducibility of the results.

### qRT-PCR

RT-qPCR was performed in quadruplicate with cDNAs generated from 1 μg of total RNA using the RT2 PCR Array First Strand Kit (QIAGEN, SABiosciences). qPCR was carried out in a 20 μL reaction volume containing RT^2^ SYBR Green qPCR Master Mix (QIAGEN, SABiosciences) and 300 nM of both forward and reverse primers recognizing a sequence of human HTT (5′-CTGCACCGACCAAAGAAAGAAC-3′ and 5′-CATAGCGATGCCCAGAAGTTTC-3′) using a Realplex thermal cycler (Eppendorf). Values for HTT mRNA were normalized to a reference, β-actin (ACTB 5′-TGAAGGTGACAGCAGTCGGTTG-3′ and 5′-GGCTTTTAGGATGGCAAGGGAC-3′), according to following formula:

$$RE\;\;Htt=\frac{2^{-Ct\;Htt}}{2^{-Ct\;\text{actin}}},$$

where RE = relative expression and Ct = cycle threshold. Data are expressed as mean values ± SEM.

### Culture, Neural Induction, and Striatal Differentiation of Human iPSCs

The human HD-iPSC line (60 CAG HD line) from the Coriell Institute for Medical Research was cultured on L7 (Lonza) matrix in STEMPRO medium (Invitrogen) supplemented with 10 ng/mL recombinant human fibroblast growth factor 2 (FGF2). Cells were fed daily and manually passaged every 5–7 days. Striatal neuron precursors were derived as previously described in Nicoleau et al.^60^ and Arber et al.^61^ Neuralized and patterned human iPSCs, enriched for ventral telencephalic progenitors, were collected after 21 DIV using Accutase for 10–20 min at 37°C and frozen in CryoStor CS10 (Invitrogen) freezing media. For terminal neuronal differentiation of striatal precursors, cells were plated on polyornithine laminin-coated dishes at 40,000 cells/cm^2^ in DMEM/F12 media supplemented with N2, B27, 20 ng/mL brain-derived neurotrophic factor (BDNF; R&D Systems), 0.5 mM N6,2′-O-dibutyryladenosine 3′,5′-cyclic monophosphate sodium salt (dbcAMP; Sigma-Aldrich), 0.5 mM valpromide (Lancaster Synthesis), and 25 ng/mL Activin A for 30–35 additional days.

### Lentiviral Transduction of Neuronal Cultures, RNA Extraction, and qRT-PCR

Neuronal cultures were transduced 15 days after plating by adding 15 ng of p24 viral particles per 100,000 neurons in the culture media. Fifteen days after transduction, infected neuronal cultures were processed for total RNA extraction using the RNeasy Plus Mini Kit (QIAGEN). The purified RNA was quantified with a NanoDrop ND-1000A spectrophotometer. Transcription was performed on 500 ng of RNA using the Cloned AMV First-Strand cDNA Synthesis Kit (Invitrogen). cDNA synthesis was performed using total RNA primed with 50 μM oligo(dT) mixed with 50 ng/μL random hexamers according to the manufacturer’s protocol. qRT-PCR was performed using Power SYBR Green PCR Mix and an LC480 system (Roche). Quantification was performed at a threshold detection limit (Ct value). The Ct of each target gene was normalized to the 18S housekeeping gene (18S-FW: 5′-GAGGATGAGGTGGAACGTGT-3′; 18S-RV: 5′-TCTTCAGTCGCTCCAGGTCT-3′; HTT-FW: 5′-GCTGCTGGTTGGACAGAAACTC-3′; HTT-RV: 5′-AGTGATTGTTGCTATGGAGCGG-3′). HTT mRNA allelic ratio analysis was performed using TaqMan SNP Genotyping Assays (Hs00918153_m1; Applied Biosystems) on the same cDNA used for qRT-PCR, as previously described.^73^ The median of the difference of the Ct of each target allele (three technical replicates) was used to calculate the HTT allelic ratio expressed as the percentage of rs362331 SNP “C” and “T” in all HTT mRNA (genomic DNA was used as a control sample with a 50/50 HTT allelic ratio).^74^

### RNA-Seq Analyses

For each of the six samples, mRNA was purified from 1 μg of total RNA using the Dynabeads mRNA DIRECT Micro kit. The RNA was fragmented, reverse transcribed, and barcoded using the Ion Total RNA-Seq kit v2 and the Ion Xpress RNA-Seq barcode kit, following the protocol of the manufacturer (Thermo Fisher). The amplicons were quantified using the Agilent High Sensitivity DNA kit before the samples were pooled into sets of three. Emulsion PCR and Enrichment was performed on the Ion One Touch Instrument (Ion ES) using the Ion PI Template OT2 200 kit v3 (Thermo Fisher). Samples were manually loaded on an Ion PI v3 chip and sequenced on the Ion Proton System using Ion PI Sequencing 200 Kit v3 chemistry (200 bp read length; Life Technologies). The Ion Proton reads (FASTQ files) were imported into the RNA-seq pipeline of Partek Flow software (v 4.0; Partek) using hg19 as a reference genome. To determine differentially expressed genes between groups, we quantified mapped reads using the Partek E/M algorithm, and differentially expressed genes were identified using the Partek Gene Specific Analysis (GSA) algorithm. Biological interpretations of the list of differentially expressed genes were generated using Partek Genomics Suite (v6.6) and Gene Set Enrichment Analysis (GSEA) software from the Broad Institute (http://www.broadinstitute.org/gsea) or online (EnrichR).

### Illumina Sequencing of Small RNAs

The mirVana miRNA Isolation kit (Life Technologies) was used to extract total RNA from infected HEK293T cells. Shortly after washing with PBS, cells were disrupted with a Lysis/binding buffer before proceeding to organic extraction using acid-phenol-chloroform. The aqueous phase was recovered and the RNA isolated using 100% ethanol, the lysate-ethanol mix loaded onto a filter cartridge, the tubes centrifuged, and the filters washed three times before final elution with nuclease-free water (Thermo Fisher). RNAs were stored at −80°C. Total RNA (4 μg) was then size separated on a 15% TBE (Tris-base, boric acid, EDTA)-urea gel (Thermo Fisher Scientific). The small RNA fraction was recovered from the gel and used in subsequent library preparation steps. Libraries of small RNAs for sequencing were prepared using NEXTflex Small RNA Sequencing Kit v2 reagents (BIOO Scientific) according to the protocol supplied by the manufacturer and 4 μg of total RNA. Cluster generation was performed with the resulting libraries using Illumina TruSeq SR Cluster Kit v4 reagents (catalog no. GD-401-4001; Illumina) and sequenced on the Illumina HiSeq 2500 using TruSeq SBS Kit V4 reagents (catalog no. FC-401-4002). Sequencing data were processed using Illumina Pipeline Software, version 1.84. NGS small RNA raw datasets were analyzed using CLC Genomics Workbench 8 (QIAGEN). The reads were first adaptor-trimmed using the CLC setting: minus strand, 5′-CCTTGGCACCCGAGAATTCCA-3′. The first and last four bases (4N) were then clipped from the adaptor-trimmed reads. All reads containing ambiguity N symbols and reads shorter than 15 nt, longer than 55 nt, or represented less than 10 times were discarded. The obtained unique small RNA reads were aligned to the reference sequence of the siHtt6-19nt construct with a maximum of 3 nt mismatches allowed. The percentages of reads matching the siHtt6-19nt reference sequence based on the total number of small RNA reads were calculated.

### In Vivo Experiments

Adult 20 g male C57/BL6 mice were used (Iffa Credo, Charles River) for all in vivo experiments. The animals were housed in a temperature-controlled room and maintained on a 12 hr day/night cycle. Food and water were available ad libitum. The animal facility was approved by veterinarian inspectors (authorization no. VD-H18) and complies with Swiss regulations concerning the care and use of laboratory animals (authorization no. 2782, 2888, and 3073). The experiments were performed in accordance with the European Community directive (86/609/EEC) for the care and use of laboratory animals.

### Stereotaxic Injections of LVs

Concentrated viral stocks were thawed on ice and resuspended by repeated pipetting. The mice were anesthetized using 75 mg/kg ketamine and 10 mg/kg xylazine, administered intraperitoneally. LVs were stereotaxically injected into the striatum using a 34-gauge blunt-tip needle linked to a Hamilton syringe (Hamilton) by a polyethylene catheter at the following stereotaxic coordinates: 0.5 mm rostral to bregma, 2 mm lateral to midline, and 3.5 mm from the skull surface. Each mouse received vectors encoding HTT and a control siRNA LV targeting luciferase mRNA in the left striatum and vectors encoding both HTT and siRNA targeting HTT in the right striatum.

Each viral vector (200 ng of p24 antigen) was injected at a rate of 0.2 μL/min using an automatic injector (Stoelting) and the needle was left in place for an additional 5 min. The skin was closed using 4-0 Prolene sutures (Ethicon, Johnson and Johnson) for mice.

### Histological Processing

Eight weeks after lentiviral injection, animals received an overdose of sodium pentobarbital and were transcardially perfused with PBS followed by 4% paraformaldehyde (Fluka; Sigma) and 10% picric acid fixation. Brains were removed and post-fixed in 4% paraformaldehyde and 10% picric acid for 24 hr and then cryoprotected in 30% sucrose, 0.1 M PBS for 48 hr. A sledge microtome with a freezing stage at −25°C (SM2400; Leica Microsystems AG) was used to cut coronal brain sections of 25 μm thickness. Sections throughout the entire striatum were collected and stored free-floating in PBS supplemented with 0.12 μM sodium azide in 96-well plates at 4°C.

Striatal sections from injected mice were processed for immunohistochemistry for dopamine and cAMP-regulated phosphoprotein with a molecular mass of 32 kDa (DARPP-32, rabbit antibody SC11365; Santa Cruz Biotechnology) and Ubi (rabbit antibody Z0458; Dakocytomation) following the same protocol. Sections were pre-incubated for 1 hr in phenylhydrazine (107251; Merck KGaA) diluted 1/1,000 in 0.1 M PBS at 37°C. They were rinsed three times in 0.1 M PBS and incubated for 1 hr in a blocking solution of 10% normal goat serum-0.1% Triton X-100 in 0.1 M PBS. Sections were incubated overnight at 4°C in a solution containing the first antibody diluted 1/1,000 (in the blocking solution for Ubi and in 0.1 M PBS-5% normal goat serum for DARPP-32). They were washed three times with PBS before applying the secondary antibody diluted 1/200 in PBS-1% normal goat serum (biotinylated goat anti-rabbit, BA1000; Vector Laboratories) for 1 hr at room temperature. The complex was revealed using the Vectastain ABC kit (PK-6100; Vector Laboratories), with 3,3′-diaminobenzidine tetrahydrochloride (DAB metal concentrate; Pierce) as substrate. The sections were mounted, dehydrated by soaking twice in 100% ethanol/toluene, and coverslips applied in Eukitt (O. Kindler).

### Quantification of DARPP-32 Lesions

The loss of DARPP-32 expression was analyzed by collecting digitized images of approximately 12 sections per animal (150 μm between sections) with a slide scanner and quantifying the areas of the lesions in square millimeters (mm^2^) using image analysis software (MCID Core 7.0, InterFocus Imaging; GE Healthcare Niagara). Lesion areas in each section were defined as regions poor in DARPP-32 staining relative to the surrounding tissue. The volume was then estimated using the following formula: volume = d * (*a*1 + *a*2 + *a*3 …), where *d* is the distance between serial sections (25 μm), and *a*1, *a*2, *a*3, … are DARPP-32-depleted areas for individual sections. The lesion size for each animal is expressed as the total lesion volume in 8–12 sections. The lesion volume for each group is expressed as the mean ± SEM of individual values. Statistical analysis was performed using a Wilcoxon test for paired samples (Statistica 5.1; Statsoft). The significance level was set at p < 0.05.

### Quantification of Inclusion Formation

For estimation of the number of Ubi-positive HTT inclusions, 12 coronal sections of the striatum (separated by 150 μm) were scanned with a 10× objective using a Zeiss Axioplan2 imaging microscope equipped with an automated motorized stage and acquisition system (Mercator Pro V6.50; ExploraNova). The quantification of all Ubi-positive objects with an apparent cross-sectional area between 1 and 50 μm^2^ was performed as previously reported.^17^ The number of Ubi-positive aggregates for each group is expressed as the mean ± SEM of individual values for each mouse. Statistical analysis was performed using a Wilcoxon test for paired samples (Statistica 5.1; Statsoft). The significance level was set at p < 0.05.

### PCR Array Analysis of Inflammatory Gene Expression in Tissue Samples

We designed custom PCR arrays to characterize the changes of expression of genes involved in the detection of double-stranded RNA and their effectors, genes involved in inflammation pathways (Table 2), and markers of the cell-mediated immune response either 24 hr or 6 weeks after bilateral injection of 100 or 200 ng of purified LVs or 200 ng of non-purified LVs in the striatum. An additional mouse injected bilaterally with a high dose of shHTT6 (1,000 ng) served as a positive control of inflammation at 24 hr.

Mice were sacrificed by pentobarbital overdose 24 hr or 6 weeks after bilateral infection of each siRNA construct or PBS in the striatum. Brains were dissected, placed on ice, and sliced using a mouse brain matrix into 1-mm-thick fresh sections. Striatum samples were removed from both hemispheres of two sections surrounding the injection site, quickly lysed and homogenized in TRIzol, and stored at −80°C before RNA extraction.

Reverse transcription was performed on 1 μg of RNA using the RT^2^ PCR Array First Strand cDNA Synthesis Kit (103C-03; SABiosciences). The quality of all samples was first assessed using quality-control arrays to verify RNA integrity and the presence of inhibitors of reverse transcription and PCR amplification and genomic and general DNA contamination (PAMM-999A; SABiosciences). qRT-PCR was performed with the RT2 SYBR Green qPCR Master Mix (PA-010; SABiosciences) on our custom RT2 Profiler PCR Array (SABiosciences) using a RealPlex system (Eppendorf). The expression level of each gene was normalized to the average expression of the housekeeping genes Hsp90ab1 and GAPDH. The fold change was calculated such that the normalized gene expression [2ˆ(−Delta Ct)] in the shHTT6 samples was divided by the normalized gene expression [2ˆ(−Delta Ct)] in the PBS control samples. The p values were calculated based on the Student’s t test of the 2ˆ(−Delta Ct) values for each gene in the PBS control group versus shHHT6 LV groups, and p < 0.05 was considered to be statistically significant.

## Author Contributions

K.C., N. Déglon, P.H., and A.L.P. designed the experiments. S.M., M.-C.G., K.C., and R.H. performed the molecular biology and RT-PCR experiments. V.Z., R.H., and K.C. performed the immunohistology experiments, and G.A. performed the in vivo experimentation. B.A., J.M., M.R., and N. Dufour provided technical support for cell culture and viral vector production, and M.J. provided the bioinformatics analysis of shHTT6 synthesis and processing. K.C., N. Déglon, and A.L.P. wrote the manuscript. All authors revised the manuscript.

## Conflicts of Interest

The authors have no conflict of interest to declare.

## Supplemental Information

Table S1. List of the 181 Genes Differentially Expressed in shHTT6-Treated HD-iPSCs: Fold Change > 1.5, p < 0.01

Table S2. List of Potential Targets of the Seed Sequence of the shHTT6 in the 3′ UTR of Genes Based on the siPOTS Website

## References

[1] Huntington, G. (1872). On chorea. The Medical and Surgical Reporter: A Weekly Journal, (Philadelphia: SW Butler) *26*, 317–321.

[2] The Huntington’s Disease Collaborative Research Group (1993). A novel gene containing a trinucleotide repeat that is expanded and unstable on Huntington’s disease chromosome. Cell.

[3] Godin J.D., Colombo K., Molina-Calavita M., Keryer G., Zala D., Charrin B.C., Dietrich P., Volvert M.L., Guillemot F., Dragatsis I. (2010). Huntingtin is required for mitotic spindle orientation and mammalian neurogenesis. Neuron.

[4] Nguyen G.D., Gokhan S., Molero A.E., Mehler M.F. (2013). Selective roles of normal and mutant huntingtin in neural induction and early neurogenesis. PLoS ONE.

[5] Rigamonti D., Bauer J.H., De-Fraja C., Conti L., Sipione S., Sciorati C., Clementi E., Hackam A., Hayden M.R., Li Y. (2000). Wild-type huntingtin protects from apoptosis upstream of caspase-3. J. Neurosci..

[6] Colin E., Zala D., Liot G., Rangone H., Borrell-Pagès M., Li X.J., Saudou F., Humbert S. (2008). Huntingtin phosphorylation acts as a molecular switch for anterograde/retrograde transport in neurons. EMBO J..

[7] Zala D., Hinckelmann M.V., Yu H., Lyra da Cunha M.M., Liot G., Cordelières F.P., Marco S., Saudou F. (2013). Vesicular glycolysis provides on-board energy for fast axonal transport. Cell.

[8] Wessels D., Lusche D.F., Scherer A., Kuhl S., Myre M.A., Soll D.R. (2014). Huntingtin regulates Ca(2+) chemotaxis and K(+)-facilitated cAMP chemotaxis, in conjunction with the monovalent cation/H(+) exchanger Nhe1, in a model developmental system: insights into its possible role in Huntington’s disease. Dev. Biol..

[9] Saudou F., Humbert S. (2016). The biology of Huntingtin. Neuron.

[10] Hocaoglu M.B., Gaffan E.A., Ho A.K. (2012). The Huntington’s Disease health-related Quality of Life questionnaire (HDQoL): a disease-specific measure of health-related quality of life. Clin. Genet..

[11] Clay E., De Nicola A., Dorey J., Squitieri F., Aballéa S., Martino T., Tedroff J., Zielonka D., Auquier P., Verny C., Toumi M. (2012). Validation of the first quality-of-life measurement for patients with Huntington’s disease: the Huntington Quality of Life Instrument. Int. Clin. Psychopharmacol..

[12] Read J., Jones R., Owen G., Leavitt B.R., Coleman A., Roos R.A., Dumas E.M., Durr A., Justo D., Say M., TRACK-HD investigators (2013). Quality of life in Huntington’s disease: a comparative study investigating the impact for those with pre-manifest and early manifest disease, and their partners. J. Huntingtons Dis..

[13] Zielonka D., Mielcarek M., Landwehrmeyer G.B. (2015). Update on Huntington’s disease: advances in care and emerging therapeutic options. Parkinsonism Relat. Disord..

[14] Harper S.Q., Staber P.D., He X., Eliason S.L., Martins I.H., Mao Q., Yang L., Kotin R.M., Paulson H.L., Davidson B.L. (2005). RNA interference improves motor and neuropathological abnormalities in a Huntington’s disease mouse model. Proc. Natl. Acad. Sci. USA.

[15] Rodriguez-Lebron E., Denovan-Wright E.M., Nash K., Lewin A.S., Mandel R.J. (2005). Intrastriatal rAAV-mediated delivery of anti-huntingtin shRNAs induces partial reversal of disease progression in R6/1 Huntington’s disease transgenic mice. Mol. Ther..

[16] Franich N.R., Fitzsimons H.L., Fong D.M., Klugmann M., During M.J., Young D. (2008). AAV vector-mediated RNAi of mutant huntingtin expression is neuroprotective in a novel genetic rat model of Huntington’s disease. Mol. Ther..

[17] Drouet V., Perrin V., Hassig R., Dufour N., Auregan G., Alves S., Bonvento G., Brouillet E., Luthi-Carter R., Hantraye P., Déglon N. (2009). Sustained effects of nonallele-specific Huntingtin silencing. Ann. Neurol..

[18] Nasir J., Floresco S.B., O’Kusky J.R., Diewert V.M., Richman J.M., Zeisler J., Borowski A., Marth J.D., Phillips A.G., Hayden M.R. (1995). Targeted disruption of the Huntington’s disease gene results in embryonic lethality and behavioral and morphological changes in heterozygotes. Cell.

[19] Duyao M.P., Auerbach A.B., Ryan A., Persichetti F., Barnes G.T., McNeil S.M., Ge P., Vonsattel J.P., Gusella J.F., Joyner A.L. (1995). Inactivation of the mouse Huntington’s disease gene homolog Hdh. Science.

[20] Zeitlin S., Liu J.P., Chapman D.L., Papaioannou V.E., Efstratiadis A. (1995). Increased apoptosis and early embryonic lethality in mice nullizygous for the Huntington’s disease gene homologue. Nat. Genet..

[21] Wang G., Liu X., Gaertig M.A., Li S., Li X.J. (2016). Ablation of huntingtin in adult neurons is nondeleterious but its depletion in young mice causes acute pancreatitis. Proc. Natl. Acad. Sci. USA.

[22] DiFiglia M., Sena-Esteves M., Chase K., Sapp E., Pfister E., Sass M., Yoder J., Reeves P., Pandey R.K., Rajeev K.G. (2007). Therapeutic silencing of mutant huntingtin with siRNA attenuates striatal and cortical neuropathology and behavioral deficits. Proc. Natl. Acad. Sci. USA.

[23] Boudreau R.L., Martins I., Davidson B.L. (2009). Artificial microRNAs as siRNA shuttles: improved safety as compared to shRNAs in vitro and in vivo. Mol. Ther..

[24] Godinho B.M., Malhotra M., O’Driscoll C.M., Cryan J.F. (2015). Delivering a disease-modifying treatment for Huntington’s disease. Drug Discov. Today.

[25] Whitehead K.A., Langer R., Anderson D.G. (2009). Knocking down barriers: advances in siRNA delivery. Nat. Rev. Drug Discov..

[26] Brummelkamp T.R., Bernards R., Agami R. (2002). A system for stable expression of short interfering RNAs in mammalian cells. Science.

[27] Hu X., Hipolito S., Lynn R., Abraham V., Ramos S., Wong-Staal F. (2004). Relative gene-silencing efficiencies of small interfering RNAs targeting sense and antisense transcripts from the same genetic locus. Nucleic Acids Res..

[28] Li L., Lin X., Khvorova A., Fesik S.W., Shen Y. (2007). Defining the optimal parameters for hairpin-based knockdown constructs. RNA.

[29] Vlassov A.V., Korba B., Farrar K., Mukerjee S., Seyhan A.A., Ilves H., Kaspar R.L., Leake D., Kazakov S.A., Johnston B.H. (2007). shRNAs targeting hepatitis C: effects of sequence and structural features, and comparision with siRNA. Oligonucleotides.

[30] Arvey A., Larsson E., Sander C., Leslie C.S., Marks D.S. (2010). Target mRNA abundance dilutes microRNA and siRNA activity. Mol. Syst. Biol..

[31] Larsson C., Grundberg I., Söderberg O., Nilsson M. (2010). In situ detection and genotyping of individual mRNA molecules. Nat. Methods.

[32] Hong S.W., Jiang Y., Kim S., Li C.J., Lee D.K. (2014). Target gene abundance contributes to the efficiency of siRNA-mediated gene silencing. Nucleic Acid Ther..

[33] Larsson E., Sander C., Marks D. (2010). mRNA turnover rate limits siRNA and microRNA efficacy. Mol. Syst. Biol..

[34] Holen T., Amarzguioui M., Wiiger M.T., Babaie E., Prydz H. (2002). Positional effects of short interfering RNAs targeting the human coagulation trigger Tissue Factor. Nucleic Acids Res..

[35] Bohula E.A., Salisbury A.J., Sohail M., Playford M.P., Riedemann J., Southern E.M., Macaulay V.M. (2003). The efficacy of small interfering RNAs targeted to the type 1 insulin-like growth factor receptor (IGF1R) is influenced by secondary structure in the IGF1R transcript. J. Biol. Chem..

[36] Khvorova A., Reynolds A., Jayasena S.D. (2003). Functional siRNAs and miRNAs exhibit strand bias. Cell.

[37] Kretschmer-Kazemi Far R., Sczakiel G. (2003). The activity of siRNA in mammalian cells is related to structural target accessibility: a comparison with antisense oligonucleotides. Nucleic Acids Res..

[38] Overhoff M., Alken M., Far R.K., Lemaitre M., Lebleu B., Sczakiel G., Robbins I. (2005). Local RNA target structure influences siRNA efficacy: a systematic global analysis. J. Mol. Biol..

[39] McIntyre G.J., Yu Y.H., Lomas M., Fanning G.C. (2011). The effects of stem length and core placement on shRNA activity. BMC Mol. Biol..

[40] Xia H., Mao Q., Eliason S.L., Harper S.Q., Martins I.H., Orr H.T., Paulson H.L., Yang L., Kotin R.M., Davidson B.L. (2004). RNAi suppresses polyglutamine-induced neurodegeneration in a model of spinocerebellar ataxia. Nat. Med..

[41] Chen Z.J., Kren B.T., Wong P.Y., Low W.C., Steer C.J. (2005). Sleeping Beauty-mediated down-regulation of huntingtin expression by RNA interference. Biochem. Biophys. Res. Commun..

[42] Huang B., Kochanek S. (2005). Adenovirus-mediated silencing of huntingtin expression by shRNA. Hum. Gene Ther..

[43] Wang Y.L., Liu W., Wada E., Murata M., Wada K., Kanazawa I. (2005). Clinico-pathological rescue of a model mouse of Huntington’s disease by siRNA. Neurosci. Res..

[44] Machida Y., Okada T., Kurosawa M., Oyama F., Ozawa K., Nukina N. (2006). rAAV-mediated shRNA ameliorated neuropathology in Huntington disease model mouse. Biochem. Biophys. Res. Commun..

[45] Gary D.S., Davidson A., Milhavet O., Slunt H., Borchelt D.R. (2007). Investigation of RNA interference to suppress expression of full-length and fragment human huntingtin. Neuromolecular Med..

[46] Huang B., Schiefer J., Sass C., Landwehrmeyer G.B., Kosinski C.M., Kochanek S. (2007). High-capacity adenoviral vector-mediated reduction of huntingtin aggregate load in vitro and in vivo. Hum. Gene Ther..

[47] McBride J.L., Boudreau R.L., Harper S.Q., Staber P.D., Monteys A.M., Martins I., Gilmore B.L., Burstein H., Peluso R.W., Polisky B. (2008). Artificial miRNAs mitigate shRNA-mediated toxicity in the brain: implications for the therapeutic development of RNAi. Proc. Natl. Acad. Sci. USA.

[48] Boudreau R.L., McBride J.L., Martins I., Shen S., Xing Y., Carter B.J., Davidson B.L. (2009). Nonallele-specific silencing of mutant and wild-type huntingtin demonstrates therapeutic efficacy in Huntington’s disease mice. Mol. Ther..

[49] Drouet V., Ruiz M., Zala D., Feyeux M., Auregan G., Cambon K., Troquier L., Carpentier J., Aubert S., Merienne N. (2014). Allele-specific silencing of mutant huntingtin in rodent brain and human stem cells. PLoS ONE.

[50] Grimm D. (2011). The dose can make the poison: lessons learned from adverse in vivo toxicities caused by RNAi overexpression. Silence.

[51] Marques J.T., Devosse T., Wang D., Zamanian-Daryoush M., Serbinowski P., Hartmann R., Fujita T., Behlke M.A., Williams B.R. (2006). A structural basis for discriminating between self and nonself double-stranded RNAs in mammalian cells. Nat. Biotechnol..

[52] Judge A., MacLachlan I. (2008). Overcoming the innate immune response to small interfering RNA. Hum. Gene Ther..

[53] Bennasser Y., Chable-Bessia C., Triboulet R., Gibbings D., Gwizdek C., Dargemont C., Kremer E.J., Voinnet O., Benkirane M. (2011). Competition for XPO5 binding between Dicer mRNA, pre-miRNA and viral RNA regulates human Dicer levels. Nat. Struct. Mol. Biol..

[54] Grimm D., Wang L., Lee J.S., Schürmann N., Gu S., Börner K., Storm T.A., Kay M.A. (2010). Argonaute proteins are key determinants of RNAi efficacy, toxicity, and persistence in the adult mouse liver. J. Clin. Invest..

[55] McManus M.T., Petersen C.P., Haines B.B., Chen J., Sharp P.A. (2002). Gene silencing using micro-RNA designed hairpins. RNA.

[56] Harborth J., Elbashir S.M., Vandenburgh K., Manninga H., Scaringe S.A., Weber K., Tuschl T. (2003). Sequence, chemical, and structural variation of small interfering RNAs and short hairpin RNAs and the effect on mammalian gene silencing. Antisense Nucleic Acid Drug Dev..

[57] Dull T., Zufferey R., Kelly M., Mandel R.J., Nguyen M., Trono D., Naldini L. (1998). A third-generation lentivirus vector with a conditional packaging system. J. Virol..

[58] Christodoulou I., Patsali P., Stephanou C., Antoniou M., Kleanthous M., Lederer C.W. (2016). Measurement of lentiviral vector titre and copy number by cross-species duplex quantitative PCR. Gene Ther..

[59] Perrier A., Peschanski M. (2012). How can human pluripotent stem cells help decipher and cure Huntington’s disease?. Cell Stem Cell.

[60] Nicoleau C., Varela C., Bonnefond C., Maury Y., Bugi A., Aubry L., Viegas P., Bourgois-Rocha F., Peschanski M., Perrier A.L. (2013). Embryonic stem cells neural differentiation qualifies the role of Wnt/β-Catenin signals in human telencephalic specification and regionalization. Stem Cells.

[61] Arber C., Precious S.V., Cambray S., Risner-Janiczek J.R., Kelly C., Noakes Z., Fjodorova M., Heuer A., Ungless M.A., Rodríguez T.A. (2015). Activin A directs striatal projection neuron differentiation of human pluripotent stem cells. Development.

[62] Arlotta P., Molyneaux B.J., Jabaudon D., Yoshida Y., Macklis J.D. (2008). Ctip2 controls the differentiation of medium spiny neurons and the establishment of the cellular architecture of the striatum. J. Neurosci..

[63] Sussel L., Marin O., Kimura S., Rubenstein J.L. (1999). Loss of Nkx2.1 homeobox gene function results in a ventral to dorsal molecular respecification within the basal telencephalon: evidence for a transformation of the pallidum into the striatum. Development.

[64] Du T., Xu Q., Ocbina P.J., Anderson S.A. (2008). NKX2.1 specifies cortical interneuron fate by activating Lhx6. Development.

[65] Hevner R.F., Shi L., Justice N., Hsueh Y., Sheng M., Smiga S., Bulfone A., Goffinet A.M., Campagnoni A.T., Rubenstein J.L. (2001). Tbr1 regulates differentiation of the preplate and layer 6. Neuron.

[66] Leone D.P., Srinivasan K., Chen B., Alcamo E., McConnell S.K. (2008). The determination of projection neuron identity in the developing cerebral cortex. Curr. Opin. Neurobiol..

[67] Bedogni F., Hodge R.D., Elsen G.E., Nelson B.R., Daza R.A., Beyer R.P., Bammler T.K., Rubenstein J.L., Hevner R.F. (2010). Tbr1 regulates regional and laminar identity of postmitotic neurons in developing neocortex. Proc. Natl. Acad. Sci. USA.

[68] McKenna W.L., Betancourt J., Larkin K.A., Abrams B., Guo C., Rubenstein J.L., Chen B. (2011). Tbr1 and Fezf2 regulate alternate corticofugal neuronal identities during neocortical development. J. Neurosci..

[69] Han W., Kwan K.Y., Shim S., Lam M.M., Shin Y., Xu X., Zhu Y., Li M., Sestan N. (2011). TBR1 directly represses Fezf2 to control the laminar origin and development of the corticospinal tract. Proc. Natl. Acad. Sci. USA.

[70] Ferland R.J., Cherry T.J., Preware P.O., Morrisey E.E., Walsh C.A. (2003). Characterization of Foxp2 and Foxp1 mRNA and protein in the developing and mature brain. J. Comp. Neurol..

[71] Tamura S., Morikawa Y., Iwanishi H., Hisaoka T., Senba E. (2004). Foxp1 gene expression in projection neurons of the mouse striatum. Neuroscience.

[72] Ouimet C.C., Langley-Gullion K.C., Greengard P. (1998). Quantitative immunocytochemistry of DARPP-32-expressing neurons in the rat caudatoputamen. Brain Res..

[73] Lopes C., Aubert S., Bourgois-Rocha F., Barnat M., Rego A.C., Déglon N., Perrier A.L., Humbert S. (2016). Dominant-negative effects of adult-onset Huntingtin mutations alter the division of human embryonic stem cells-derived neural cells. PLoS ONE.

[74] Kay C., Collins J.A., Skotte N.H., Southwell A.L., Warby S.C., Caron N.S., Doty C.N., Nguyen B., Griguoli A., Ross C.J. (2015). Huntingtin haplotypes provide prioritized target panels for allele-specific silencing in Huntington disease patients of European ancestry. Mol. Ther..

[75] Rose S.D., Kim D.H., Amarzguioui M., Heidel J.D., Collingwood M.A., Davis M.E., Rossi J.J., Behlke M.A. (2005). Functional polarity is introduced by Dicer processing of short substrate RNAs. Nucleic Acids Res..

[76] Zhou J., Song M.S., Jacobi A.M., Behlke M.A., Wu X., Rossi J.J. (2012). Deep sequencing analyses of DsiRNAs reveal the influence of 3′ terminal overhangs on Dicing polarity, strand selectivity, and RNA editing of siRNAs. Mol. Ther. Nucleic Acids.

[77] Dallas A., Ilves H., Ge Q., Kumar P., Shorenstein J., Kazakov S.A., Cuellar T.L., McManus M.T., Behlke M.A., Johnston B.H. (2012). Right- and left-loop short shRNAs have distinct and unusual mechanisms of gene silencing. Nucleic Acids Res..

[78] Amarzguioui M., Prydz H. (2004). An algorithm for selection of functional siRNA sequences. Biochem. Biophys. Res. Commun..

[79] Naito Y., Yamada T., Ui-Tei K., Morishita S., Saigo K. (2004). siDirect: highly effective, target-specific siRNA design software for mammalian RNA interference. Nucleic Acids Res..

[80] Reynolds A., Leake D., Boese Q., Scaringe S., Marshall W.S., Khvorova A. (2004). Rational siRNA design for RNA interference. Nat. Biotechnol..

[81] Naito Y., Yoshimura J., Morishita S., Ui-Tei K. (2009). siDirect 2.0: updated software for designing functional siRNA with reduced seed-dependent off-target effect. BMC Bioinformatics.

[82] Boudreau R.L., Spengler R.M., Hylock R.H., Kusenda B.J., Davis H.A., Eichmann D.A., Davidson B.L. (2013). siSPOTR: a tool for designing highly specific and potent siRNAs for human and mouse. Nucleic Acids Res..

[83] Grimm D., Streetz K.L., Jopling C.L., Storm T.A., Pandey K., Davis C.R., Marion P., Salazar F., Kay M.A. (2006). Fatality in mice due to oversaturation of cellular microRNA/short hairpin RNA pathways. Nature.

[84] An D.S., Qin F.X., Auyeung V.C., Mao S.H., Kung S.K., Baltimore D., Chen I.S. (2006). Optimization and functional effects of stable short hairpin RNA expression in primary human lymphocytes via lentiviral vectors. Mol. Ther..

[85] Bauer M., Kinkl N., Meixner A., Kremmer E., Riemenschneider M., Förstl H., Gasser T., Ueffing M. (2009). Prevention of interferon-stimulated gene expression using microRNA-designed hairpins. Gene Ther..

[86] Hutson T.H., Foster E., Dawes J.M., Hindges R., Yáñez-Muñoz R.J., Moon L.D. (2012). Lentiviral vectors encoding short hairpin RNAs efficiently transduce and knockdown LINGO-1 but induce an interferon response and cytotoxicity in central nervous system neurones. J. Gene Med..

[87] Hutson T.H., Foster E., Moon L.D., Yáñez-Muñoz R.J. (2014). Lentiviral vector-mediated RNA silencing in the central nervous system. Hum. Gene Ther. Methods.

[88] Lafon M., Megret F., Lafage M., Prehaud C. (2006). The innate immune facet of brain: human neurons express TLR-3 and sense viral dsRNA. J. Mol. Neurosci..

[89] Bridge A.J., Pebernard S., Ducraux A., Nicoulaz A.L., Iggo R. (2003). Induction of an interferon response by RNAi vectors in mammalian cells. Nat. Genet..

[90] Ge Q., Ilves H., Dallas A., Kumar P., Shorenstein J., Kazakov S.A., Johnston B.H. (2010). Minimal-length short hairpin RNAs: the relationship of structure and RNAi activity. RNA.

[91] Cifuentes D., Xue H., Taylor D.W., Patnode H., Mishima Y., Cheloufi S., Ma E., Mane S., Hannon G.J., Lawson N.D. (2010). A novel miRNA processing pathway independent of Dicer requires Argonaute2 catalytic activity. Science.

[92] Cheloufi S., Dos Santos C.O., Chong M.M., Hannon G.J. (2010). A dicer-independent miRNA biogenesis pathway that requires Ago catalysis. Nature.

[93] Yang J.S., Maurin T., Lai E.C. (2012). Functional parameters of Dicer-independent microRNA biogenesis. RNA.

[94] Yang J.S., Maurin T., Robine N., Rasmussen K.D., Jeffrey K.L., Chandwani R., Papapetrou E.P., Sadelain M., O’Carroll D., Lai E.C. (2010). Conserved vertebrate mir-451 provides a platform for Dicer-independent, Ago2-mediated microRNA biogenesis. Proc. Natl. Acad. Sci. USA.

[95] Ma H., Zhang J., Wu H. (2014). Designing Ago2-specific siRNA/shRNA to avoid competition with endogenous miRNAs. Mol. Ther. Nucleic Acids.

[96] Liu Y.P., Schopman N.C., Berkhout B. (2013). Dicer-independent processing of short hairpin RNAs. Nucleic Acids Res..

[97] Martin J.N., Wolken N., Brown T., Dauer W.T., Ehrlich M.E., Gonzalez-Alegre P. (2011). Lethal toxicity caused by expression of shRNA in the mouse striatum: implications for therapeutic design. Gene Ther..

[98] Ehlert E.M., Eggers R., Niclou S.P., Verhaagen J. (2010). Cellular toxicity following application of adeno-associated viral vector-mediated RNA interference in the nervous system. BMC Neurosci..

[99] Cuccato G., Polynikis A., Siciliano V., Graziano M., di Bernardo M., di Bernardo D. (2011). Modeling RNA interference in mammalian cells. BMC Syst. Biol..

[100] Kalathur R.K., Hernández-Prieto M.A., Futschik M.E. (2012). Huntington’s disease and its therapeutic target genes: a global functional profile based on the HD Research Crossroads database. BMC Neurol..

[101] Valenza M., Rigamonti D., Goffredo D., Zuccato C., Fenu S., Jamot L., Strand A., Tarditi A., Woodman B., Racchi M. (2005). Dysfunction of the cholesterol biosynthetic pathway in Huntington’s disease. J. Neurosci..

[102] Li P., Nie Y., Yu J. (2015). An effective method to identify shared pathways and common factors among neurodegenerative diseases. PLoS ONE.

[103] Elias S., McGuire J.R., Yu H., Humbert S. (2015). Huntingtin is required for epithelial polarity through RAB11A-mediated apical trafficking of PAR3-aPKC. PLoS Biol..

[104] Olejniczak M., Urbanek M.O., Jaworska E., Witucki L., Szczesniak M.W., Makalowska I., Krzyzosiak W.J. (2016). Sequence-non-specific effects generated by various types of RNA interference triggers. Biochim. Biophys. Acta.

[105] Pebernard S., Iggo R.D. (2004). Determinants of interferon-stimulated gene induction by RNAi vectors. Differentiation.

[106] McBride J.L., Pitzer M.R., Boudreau R.L., Dufour B., Hobbs T., Ojeda S.R., Davidson B.L. (2011). Preclinical safety of RNAi-mediated HTT suppression in the rhesus macaque as a potential therapy for Huntington’s disease. Mol. Ther..

[107] Grondin R., Kaytor M.D., Ai Y., Nelson P.T., Thakker D.R., Heisel J., Weatherspoon M.R., Blum J.L., Burright E.N., Zhang Z., Kaemmerer W.F. (2012). Six-month partial suppression of Huntingtin is well tolerated in the adult rhesus striatum. Brain.

[108] Lombardi M.S., Jaspers L., Spronkmans C., Gellera C., Taroni F., Di Maria E., Donato S.D., Kaemmerer W.F. (2009). A majority of Huntington’s disease patients may be treatable by individualized allele-specific RNA interference. Exp. Neurol..

[109] Monteys A.M., Ebanks S.A., Keiser M.S., Davidson B.L. (2017). CRISPR/Cas9 editing of the mutant Huntingtin allele in vitro and in vivo. Mol. Ther..

[110] Shin J.W., Kim K.H., Chao M.J., Atwal R.S., Gillis T., MacDonald M.E., Gusella J.F., Lee J.M. (2016). Permanent inactivation of Huntington’s disease mutation by personalized allele-specific CRISPR/Cas9. Hum. Mol. Genet..

[111] Chung D.W., Rudnicki D.D., Yu L., Margolis R.L. (2011). A natural antisense transcript at the Huntington’s disease repeat locus regulates HTT expression. Hum. Mol. Genet..

[112] Palfi S., Brouillet E., Jarraya B., Bloch J., Jan C., Shin M., Condé F., Li X.J., Aebischer P., Hantraye P., Déglon N. (2007). Expression of mutated huntingtin fragment in the putamen is sufficient to produce abnormal movement in non-human primates. Mol. Ther..

[113] Hirano M., Kato S., Kobayashi K., Okada T., Yaginuma H., Kobayashi K. (2013). Highly efficient retrograde gene transfer into motor neurons by a lentiviral vector pseudotyped with fusion glycoprotein. PLoS ONE.

[114] Bankiewicz K.S., Sudhakar V., Samaranch L., San Sebastian W., Bringas J., Forsayeth J. (2016). AAV viral vector delivery to the brain by shape-conforming MR-guided infusions. J. Control. Release.

[115] Hintiryan H., Foster N.N., Bowman I., Bay M., Song M.Y., Gou L., Yamashita S., Bienkowski M.S., Zingg B., Zhu M. (2016). The mouse cortico-striatal projectome. Nat. Neurosci..

[116] Follenzi A., Ailles L.E., Bakovic S., Geuna M., Naldini L. (2000). Gene transfer by lentiviral vectors is limited by nuclear translocation and rescued by HIV-1 pol sequences. Nat. Genet..

[117] Déglon N., Tseng J.L., Bensadoun J.C., Zurn A.D., Arsenijevic Y., Pereira de Almeida L., Zufferey R., Trono D., Aebischer P. (2000). Self-inactivating lentiviral vectors with enhanced transgene expression as potential gene transfer system in Parkinson’s disease. Hum. Gene Ther..

[118] de Almeida L.P., Ross C.A., Zala D., Aebischer P., Déglon N. (2002). Lentiviral-mediated delivery of mutant huntingtin in the striatum of rats induces a selective neuropathology modulated by polyglutamine repeat size, huntingtin expression levels, and protein length. J. Neurosci..

[119] Hottinger A.F., Azzouz M., Déglon N., Aebischer P., Zurn A.D. (2000). Complete and long-term rescue of lesioned adult motoneurons by lentiviral-mediated expression of glial cell line-derived neurotrophic factor in the facial nucleus. J. Neurosci..

[120] Amendola M., Passerini L., Pucci F., Gentner B., Bacchetta R., Naldini L. (2009). Regulated and multiple miRNA and siRNA delivery into primary cells by a lentiviral platform. Mol. Ther..

[121] Makeyev A.V., Chkheidze A.N., Liebhaber S.A. (1999). A set of highly conserved RNA-binding proteins, alphaCP-1 and alphaCP-2, implicated in mRNA stabilization, are coexpressed from an intronless gene and its intron-containing paralog. J. Biol. Chem..

